# Mutant p53 blocks SESN1/AMPK/PGC-1α/UCP2 axis increasing mitochondrial O_2ˉ_· production in cancer cells

**DOI:** 10.1038/s41416-018-0288-2

**Published:** 2018-10-15

**Authors:** Marco Cordani, Giovanna Butera, Ilaria Dando, Margalida Torrens-Mas, Elena Butturini, Raffaella Pacchiana, Elisa Oppici, Chiara Cavallini, Sara Gasperini, Nicola Tamassia, Mercedes Nadal-Serrano, Michela Coan, Davide Rossi, Gianluca Gaidano, Michele Caraglia, Sofia Mariotto, Riccardo Spizzo, Pilar Roca, Jordi Oliver, Maria Teresa Scupoli, Massimo Donadelli

**Affiliations:** 1 Department of Neurosciences, Biomedicine and Movement Sciences, Section of Biochemistry, University of Verona, Verona, Italy; 2 Biochemistry Department, Universidad Autónoma de Madrid (UAM), Instituto de Investigaciones Biomédicas “Alberto Sols” (CSIC-UAM), IdiPAZ, Madrid, Spain; 3 Grupo Multidisciplinar de Oncología Traslacional, Instituto Universitario de Investigación en Ciencias de la Salud (IUNICS), Palma de Mallorca, Illes Balears, Spain; 4 Ciber Fisiopatología Obesidad y Nutrición (CB06/03), Instituto Salud Carlos III, Madrid, Spain; 5 Instituto de Investigación Sanitaria de Palma (IdISPa), Hospital Universitario Son Espases, edificio S. E-07120, Palma de Mallorca, Illes Balears, Spain; 6 Research Center LURM (Interdepartmental Laboratory of Medical Research), University of Verona, Verona, Italy; 7 Department of Medicine, Section of General Pathology, University of Verona, Verona, Italy; 8 Vall d’Hebron Institut d’Oncologia (VHIO), Barcelona, Spain; 9 CIBERONC, Madrid, Spain; 10 Division of Molecular Oncology, Department of Translational Research, CRO National Cancer Institute Aviano, Aviano, Italy; 11 Hematology, Oncology Institute of Southern Switzerland, Bellinzona, Switzerland; 12 Institute of Oncology Research, Bellinzona, Switzerland; 13 Division of Hematology, Department of Translational Medicine, University of Eastern Piedmont, Novara, Italy; 14 Department of Biochemistry, Biophysics and General Pathology, University of Campania “L. Vanvitelli”, Naples, Italy

**Keywords:** Oncogenes, Tumour-suppressor proteins

## Abstract

**Background:**

The *TP53* tumor suppressor gene is the most frequently altered gene in tumors and mutant p53 gain-of-function isoforms actively promote cancer malignancy.

**Methods:**

A panel of wild-type and mutant p53 cancer cell lines of different tissues, including pancreas, breast, skin, and lung were used, as well as chronic lymphocytic leukemia (CLL) patients with different *TP53* gene status. The effects of mutant p53 were evaluated by confocal microscopy, reactive oxygen species production assay, immunoblotting, and quantitative reverse transcription polymerase chain reaction after cellular transfection.

**Results:**

We demonstrate that oncogenic mutant p53 isoforms are able to inhibit SESN1 expression and consequently the amount of SESN1/AMPK complex, resulting in the downregulation of the AMPK/PGC-1α/UCP2 axis and mitochondrial O_2_ˉ· production. We also show a correlation between the decrease of reduced thiols with a poorer clinical outcome of CLL patients bearing mutant *TP53* gene. The restoration of the mitochondrial uncoupling protein 2 (UCP2) expression, as well as the addition of the radical scavenger *N*-acetyl-l-cysteine, reversed the oncogenic effects of mutant p53 as cellular hyper-proliferation, antiapoptotic effect, and resistance to drugs.

**Conclusions:**

The inhibition of the SESN1/AMPK/PGC-1α/UCP2 axis contributes to the pro-oxidant and oncogenic effects of mutant p53, suggesting pro-oxidant drugs as a therapeutic approach for cancer patients bearing mutant *TP53* gene.

## Background

Mutations in the *TP53* gene occur in over 50% of the human cancers and most of them are missense mutations that result in the expression of mutant isoforms of the p53 protein,^1^ which can acquire new biological properties referred as gain-of-function (GOF). In addition to the loss of the tumor suppression function of wild-type p53, GOF mutant p53 proteins contribute to the maintenance and stimulation of cancer growth through the acquisition of oncogenic functions.^2,3^ In many tumors, p53 mutations are associated with high genomic instability, poor prognosis, reduced response to chemotherapy, promotion of migration, invasion and metastasis, and accelerated tumor recurrence.^4–6^ Different models have been proposed to explain the GOF activities of mutant p53, including binding and inactivation of the p53 family members p63 and p73, modulation of the activity of a number of transcription factors, or the inactivation of DNA damage molecular sensors.^7–9^ Our group documented that DNA damaging in cancer cells by gemcitabine drug stabilized the nuclear localization of mutant p53 proteins, which in turn triggered the expression of cell cycle-related genes, resulting in hyper-proliferation effects and ultimately chemoresistance.^10^ In addition, we and others demonstrated that GOF mutant p53 isoforms can alter cancer cell metabolism,^11–14^ autophagy response to various stimuli^15,16^ and cancer microenvironment.^17,18^ This broad spectrum of molecular properties indicates that GOF mutant p53 is involved in a plethora of different cellular pathways focused on cancer progression and aggressiveness.

Mitochondrial uncoupling protein 2 (UCP2) is an anion carrier protein, which uncouples the oxidative phosphorylation (OXPHOS) from ATP production by dissipating the proton gradient generated across the mitochondrial inner membrane. This prevents the proton motive force from becoming excessive, thus decreasing the formation of mitochondrial superoxide ions (O_2_ˉ·), produced by leakage of electrons from the mitochondrial transport chain.^19^ Importantly, the UCP2-mediated dissipation of the proton gradient during OXPHOS confers an antioxidant role to mitochondrial UCP2 proteins.^20^ It is well-established that eukaryotic cells have adopted many mechanisms in order to maintain a correct balance between reactive oxygen species (ROS) generation and their elimination by ROS-scavenging activities. Dysfunction of any of these antioxidant mechanisms could lead to an increase of intracellular ROS levels and alterations in the cellular redox status, resulting in the aberrant stimulation/suppression of some crucial signaling pathways. Indeed, increased ROS production can play a role in a variety of pathological conditions, including cancer, neurodegenerative diseases, and aging.^21,22^

Recently, some studies described that, in contrast to the antioxidant role of wild-type p53, mutant p53 proteins can stimulate ROS production. However, the precise molecular mechanisms involved in this aberrant regulation of ROS by mutant p53 isoforms are still incomplete. In the present study, we report that GOF mutant p53 proteins inhibit SESN1 expression and consequently the amount of the SESN1/AMPK complex, resulting in the inhibition of AMPK signaling and of proliferator-activated receptor gamma coactivator-1 alpha (PGC-1α)/UCP2 axis. We demonstrate that AMPK/PGC-1α/UCP2 blockage is functionally involved in the pro-oxidant role of mutant p53 in cancer cells stimulating mitochondrial O_2_ˉ· production without damaging mtDNA. We also disclose that UCP2 decrease and consequent ROS increase are functionally associated to mutant p53 GOF, determining hyper-proliferation, drug chemoresistance, and antiapoptotic effects in cancer cells.

## Material and methods

### Drugs and chemicals

Gemcitabine (2’,2’-difluoro-2’-deoxycytidine; GEM) was provided by Accord Healthcare (Milan, Italy) and solubilized in sterile bi-distilled water. *N*-acetyl-l-cysteine (NAC) and CP-31398 dihydrochloride hydrate were obtained from Sigma-Aldrich (Milan, Italy) and solubilized in bi-distilled sterile water. 5-Aminoimidazole-4-carboxamide ribonucleotide (AICA-R) was provided by MERK Calbiochem (Darmstadt, Germany) and solubilized in bi-distilled sterile water. RITA [5,5’-(2,5-furandiyl)bis-2-thiophenemethanol; reactivation of p53 and induction of tumor cell apoptosis] was obtained from Sigma-Aldrich and solubilized in DMSO.

### Cell culture

Pancreatic adenocarcinoma PaCa3 (WTp53), Panc1 (mutant p53-R273H), and AsPC1 (p53-null) cell lines were grown in Roswell Park Memorial Institute medium (Life Technologies, Milan, Italy), whereas lung cancer H1299 (p53-null), melanoma A375 (WTp53), MeWo (mutant p53-E258K), breast cancer MCF7 (WTp53), SKBr3 (mutant p53-R175H), MDA-MB-468 (mutant p53-R273H), and MDA-MB-231 (mutant p53-R280K) cell lines were cultured in Dulbecco's Modified Eagle's Medium (Life Technologies). All culture media were supplemented with 10% fetal bovine serum (FBS), and 50 µg/ml gentamicin sulfate (BioWhittaker, Lonza, Bergamo, Italy). Cell lines were incubated at 37 °C with 5% CO_2_. The list of the cell lines used in this study and their p53 status are summarized in Table 1. PaCa3 cell line was kindly provided by Dr. Aldo Scarpa (University of Verona, Italy) and all the other cell lines were purchased by ATCC (Manassas, Virginia, USA). Pancreatic ductal adenocarcinoma cells were previously characterized^23^ and all the cell lines were routinely tested to confirm lack of mycoplasma infection. The clones C9 (mock) and H1 (stably expressing mutant p53-R273H) of the p53-null H1299 cells were kindly provided by Dr. Riccardo Spizzo (Centro di Riferimento Oncologico, National Cancer Institute, Aviano, Italy).

**Table 1:** Tissue of origin and p53 status of cancer cell lines

Cancer cell lines	Tissue origin	p53 status	Mutation
PaCa3	Pancreas	Wild-type	None
MCF7	Breast	Wild-type	None
A375	Skin	Wild-type	None
Panc1	Pancreas	Mutated	R273H
PaCa44	Pancreas	Mutated	C176S
MDA-MB-468	Breast	Mutated	R273H
SkBr3	Breast	Mutated	R175H
MDA-MB-231	Breast	Mutated	R280K
MeWo	Skin	Mutated	E258K
AsPC1	Pancreas	Null	Gene deleted
H1299	Lung	Null	Gene deleted

### Cell proliferation assay

Cells were seeded in 96-well plates and the day after were incubated with various compounds at the indicated conditions or transfected with the indicated constructs (see figure legends). At the end of the treatments, cell growth was measured by Crystal Violet assay (Sigma-Aldrich) according to the manufacturer’s protocol, and absorbance was measured by spectrophotometric analysis (A 595 nm).

### Liposome-mediated transient cell transfection

Cells were transfected as previously described.^15^ In brief, the ectopic expression of mutant p53 in p53-null cancer cells was carried out transfecting pcDNA3-mutp53R273H or pcDNA3-mutp53R175H expression vectors, or their relative mock vector (pcDNA3). Wild-type and mutant p53 protein expression was transiently knocked-down by transfection with pRSUPER-p53 vector or its negative control (pRSUPER), kindly provided by Dr. Agami (The Netherlands Cancer Institute, Amsterdam, Netherland). Commercial siRNA smart pool of three oligonucleotides (sip53) transiently targeting p53 (Santa Cruz Biotech, Dallas, TX, USA; sc-29435) was used in the real-time qPCR to exclude back side effects and to confirm the robustness of the data. A siGFP as non-silencing control (5′-GGCTACGTCCAGGAGCGCACC-3′) was used as negative control. The ectopic expression of wild-type or dominant negative (DN)-AMPK subunit γ2 was previously described.^24^

UCP2 silencing was carried out with a specific siRNA-UCP2 (5′-GCUAAAGUCCGGUUACAGATT-3′), and a non-targeting siRNA (5′-CAGUCGCGUUUGCGACUGG-3′) was used as negative control (Ambion, Thermo Fisher Scientific, Rockford, IL, USA). UCP2 overexpression was performed using a pCMV expression vector containing the human complementary DNA of UCP2 (OriGene Technologies, Rockville, MD, USA). Cells transfected with the empty pCMV vector were used as a negative control (mock).

Knockdown of PGC-1α expression was obtained by transfecting cells with a duplex siRNA-PGC-1α having the following sequences: 5′-ACAGUGAAUUUAAACGACAGCAGCU-3′ and 5′-AGCUGCUGUCGUUUAAAUUCACUGU-3′ purchased from Life Technologies.

### Lentivirus cell transduction

To silence mutant p53 expression, we used two different DNA plasmids pLKO.1 puro vectors encoding *TP53*-shRNAs (TRCN0000003756 or TRCN0000003753 Sigma-Aldrich) indicated as p53-SH1 or p53-SH2, respectively. As negative control we used a non-target shRNA control (SHC016; Sigma-Aldrich) indicated as p53-NT. To generate viral particles, 293FT were transfected using one of the pLKO shRNA DNA vector together with ViraPower Lentiviral Packaging Mix (pLP1, pLP2 and pLP/VSV-G) (Thermo Fisher Scientific, Eugene, OR, USA). Seventy-two hours later, viral supernatants were collected and transducing units per ml of supernatant were determined by limiting dilution titration in cells. A MOI (multiplicity of infection) of 5–1 (five transducing viral particles to one cell) was used for transducing cells using Polybrene (Sigma-Aldrich) at a final concentration of 8 μg/ml to increase transduction efficiency. Twenty-four hours after transduction, puromycin selection was started and mutant *TP53*-silenced cells were immediately used for experiments.

### RNA isolation and quantitative real-time PCR analysis

Total RNA was extracted from cells using PureLink RNA Mini Kit (Life Technologies, Milan, Italy) and reverse-transcribed at 37 °C for 50 min in the presence of random hexamers and Moloney murine leukemia virus reverse transcriptase (Life Technologies). Transcripts were measured by real-time qPCR using the SYBR Green assay (Applied Biosystems, Carlsbad, CA, USA) with a 7900 HT Fast Real-Time PCR System (Thermo Fisher). PCR analysis was carried out using specific oligonucleotides for the genes listed in Supplementary Table 1. The average of cycle threshold of each triplicate was analyzed according to the 2^-ΔΔCt^ method. GAPDH gene expression was used as endogenous control to standardize mRNA expression. All reactions were performed in triplicate from three independent experiments.

### Immunoblot analysis

Cells were lysed in the presence of phosphatase and protease inhibitors (50 mM Tris HCl pH 8, 150 mM NaCl, 1% Igepal CA-630, 0.5% Na-Doc, 0.1% sodium dodecyl sulphate (SDS), 1 mM Na_3_VO_4_, 1 mM NaF, 2.5 mM ethylenediaminetetraacetic acid (EDTA), 1 mM phenylmethylsulfonyl fluorid, and 1 × protease inhibitor cocktail). Protein extracts (50 μg/lane) were resolved on SDS-polyacrylamide gel and electro-blotted onto polyvinylidene difluoride (PVDF) membranes (Millipore, Milan, Italy). Primary antibodies against p53 (Santa Cruz Biotech, Dallas, Texas, USA, #sc-263), phospho (Ser15)p53 (Cell Signaling Technology, Danvers, MA, USA; #9286), phospho (Thr172)AMPK (Cell Signaling Technology, #2535), AMPK (Cell Signaling Technology, #2603), SESN1 (GeneTex, Irvine, CA, USA; #GTX118141), SESN2 (Santa Cruz Biotech, #sc-393195), PGC-1α (Calbiochem, #ST1202), UCP2 (Abnova, Taipei City, Taiwan; #PAB7242), GAPDH (Cell Signaling Technology, #5174 s), alpha-tubulin (Oncogene, La Jolla, CA, USA; #CP06), and secondary anti-mouse or anti-rabbit IgGs (Upstate Biotechnology, Milan, Italy) horseradish peroxidase-conjugated antibodies were used. All antibodies were diluted in 5% (w/v) non-fat milk or bovine serum albumin (BSA) in TBS–Tween. The immunocomplexes were visualized by chemiluminescent substrates (Amersham Pharmacia Biotech, Milan, Italy) using the Chemidoc XRS Imaging System (Bio-Rad Laboratories, Milan, Italy) and the intensity of the chemiluminescence response was measured by processing the image with NIH ImageJ software (http://rsb.info.nih.gov/nih-image/).

### Immunoprecipitation assay

Cells were lysed in radioimmunoprecipitation assay (RIPA) buffer (20 mM Tris HCl, pH 8.0, 150 mM NaCl, 1% Nonidet P-40, 1 mM EDTA, 10% glycerol, 100 µM NaF, 1 mM Na_3_VO_4_) supplemented with protease cocktail inhibitor. For each immunoprecipitation, 2 μg of anti-mouse AMPKα antibody (Santa Cruz Biotech, #sc-74461), anti-mouse SESN1 (PA26) antibody (Santa Cruz Biotech, #sc-376170), or mouse IgG (Santa Cruz Biotech) as control were used. Equal amounts of proteins from the clarified cell lysates were incubated overnight with antibodies at 4 °C. The immune complexes were collected by addition of protein A sepharose (Millipore), rinsed extensively with RIPA buffer and eluted in a non-reducing sample buffer (62.5 mM Tris HCl, pH 6.8, 10% glycerol, 5% SDS, 0.05% bromophenol blue). After electrophoresis on 10% SDS–PAGE, proteins were transferred to PVDF membrane and non-specific binding was blocked by incubation in 3% BSA diluted in TBS–Tween. Membranes were then probed with anti-SESN1 (Santa Cruz Biotech, #sc-376170), anti-SESN2 (Santa Cruz Biotech, #sc-393195), or with anti-AMPKα (Cell Signaling Technology, #mAb-5831). After washing, blots were incubated with anti-rabbit IgG peroxidase-conjugated antibody (Cell Signaling Technology) and protein–antibody reactions were detected with chemiluminescent detection system (Immun-Star™ WesternC™ Kit, Bio-Rad) using the ChemiDoc XRS Imaging System (Bio-Rad Laboratories).

### Apoptosis assay

Cells were seeded in 96-well plates and 24 h later were treated as indicated in figure legends. At the end of the treatments, cells were fixed with 2% paraformaldehyde in phosphate-buffered saline (PBS) for 30 min at room temperature, washed twice with PBS and stained with annexin V/FITC (Bender MedSystem, Milan, Italy) in binding buffer (10 mM HEPES/NaOH pH 7.4, 140 mM NaCl, and 2.5 mM CaCl_2_) for 10 min at room temperature in the dark. Cells were then washed with binding buffer and fluorescence was measured using a multimode plate reader (Ex 485 nm and Em 535 nm) (GENios Pro, Tecan, Milan, Italy). The values were normalized on cell proliferation by Crystal Violet assay.

### Analysis of intracellular ROS

The non-fluorescent diacetylated 2’,7’-dichlorofluorescein probe (Sigma-Aldrich), which becomes highly fluorescent upon oxidation, was used to evaluate intracellular ROS production by using a multimode plate reader (GENios Pro, Tecan), as previously described.^25^ To assess ROS production with flow cytometry, we treated cells with 10 µM CM-H_2_DCFDA (Life Technologies, #C6827). After re-suspending them in ice-cold fluorescence-activated cell sorting-buffer (1% BSA in PBS), cells were immediately analyzed by BD FACSCanto II (BD Bioscience, Franklin Lakes, NJ, USA) using the 488 nm blue laser and 530/30 nm filter. Unstained cells were used to set dichlorofluorescein (DCF) fluorescent negative and positive threshold.

To evaluate mitochondrial superoxide ion (O_2_ˉ·) production we used the non-fluorescent MitoSox Red probe (Molecular Probes, Eugene, OR, USA), as previously described.^25^ It is live-cell permeant and is rapidly and selectively targeted to mitochondria where it becomes fluorescent after oxidation by O_2_ˉ·, but not by other ROS or reactive nitrogen species.

In melanoma cells, to analyze the H_2_O_2_ production level, the Amplex Red Hydrogen Peroxide/Peroxidase Assay Kit (Molecular Probes) was used. In brief, cells were seeded in 96-well plates and at the end of cell treatments, 50 µM Amplex Red reagent and 0.1 U/mL horseradish peroxidase were diluted in Krebs-Ringer phosphate buffer and the mixture was added to the cells. Fluorescence measurement was recorded at times 0, 15, and 30 min. An FLx800 microplate fluorescence reader (Bio-Tek, Winooski, VT, USA) was used, set at Ex571 nm and Em585 nm. Values were normalized on cell proliferation by Crystal Violet assay.

### Mitochondrial membrane potential determination

Mitochondrial membrane potential (ΔΨm) was measured fluorometrically by using tetramethylrhodamine methyl ester (TMRM) probe (Thermo Fisher Scientific). In brief, melanoma cells were seeded in 96-well plates and, the next day, transfected with control or p53 siRNA. After treatment, cells were incubated with 100 nM TMRM for 15 min and fluorescence was measured in an FLx800 microplate fluorescence reader (Bio-Tek) set at Ex552 nm and Em576 nm. Values were normalized per cell proliferation determined by Crystal Violet.

### Mitochondrial DNA damage analysis

Cells were seeded in six-well plates and the next day transfected with control or p53 siRNA. After 48 h, cells were harvested with TRI Reagent® (Sigma-Aldrich) and DNA was isolated according to manufacturer’s instructions. Mitochondrial DNA (mtDNA) damage was evaluated using a qPCR approach, amplifying a GC-rich region of the mtDNA. The G nucleotide is usually the first to get oxidized, and the PCR efficiency diminishes when mtDNA damage exists. Two sets of primers were used, specified in Supplementary Table 1. One of them amplifies a short fragment near the GC region, and the other amplifies a long fragment, which includes both the short fragment and the GC region, and is more sensitive to oxidative damage. Damage in mtDNA is estimated by calculating the ratio of long fragment/short fragment crossing points.

### MitoTracker and MitoSox colocalization analysis

For live cell imaging measurements, AsPC-1 cells/chamber were seeded on a four-chamber µslide, with 13 mm glass bottom (ibidi GmbH, Germany). After 24 h cells were transfected with pcDNA3-mutp53R175H, pcDNA3-mutp53R273H or the pcDNA3 empty vector (mock) by using Lipofectamine^TM^2000 according to the manufacturer’s instructions. Forty-eight hours after transfection cells were incubated for 30 minutes with a staining solution made of MitoSox Red 1:1000 (Life Technologies) and Mitotracker Green 1:5000 (Life Technologies) in medium without FBS. Before the acquisition, the medium was replaced with a special medium without red phenol (DMEM/F12 NoPhenolRED, Life Technologies) to avoid any interference with the fluorescence signal. Cell images were captured using a confocal laser-scanning fluorescence microscope Leica SP5 (Leica Microsystem, Manheim, Germany) at × 63 magnification and processed using Adobe Photoshop and ImageJ softwares (Rasband, W.S., ImageJ, U. S. National Institute of Health, Bethesda, Maryland, USA (http://rsb.info.nih.gov/ij/, 1997–2008).

### Glutathione content quantification

The intracellular glutathione (GSH) concentration was measured by endpoint spectrophotometric titration method as previously described.^26^ In brief, treated and untreated cells were lysed by freezing and thawing in 100 mM sodium phosphate buffer, pH 7.5, containing 5 mM EDTA (KPE buffer) and after centrifugation at 16,000 rpm for 10 minutes, total protein concentration was determined by using Bradford method. The supernatants were deproteinized with 5% trichloroacetic acid. For GSH measurement, acidified clear supernatants were neutralized and buffered at pH 7.4 with 200 mM K_2_HPO_4_, pH 7.5. The reaction was then started by the addition of 60 µM Ellman’s Reagent [(5,5′-dithio-bis-(2-nitrobenzoic acid)] and increase in absorbance at 412 nm was measured until no variation in absorbance was evident. The amount of total GSH was determined by comparison with GSH standard curve.

### Chronic lymphocytic leukemia (CLL) patients

Clinical annotations of 26 patients at diagnosis were collected for time-to-first treatment (TTFT) curve analysis. Diagnosis was based on 1996 National Cancer Institute-Working Group/IWCLL and 2008 Guidelines for Diagnosis and Treatment of CLL.^27^ Patient inclusion criteria were the availability of mutational status of *TP53* gene and clinical annotation and the lack of treatment prior to sample collection. Moreover, inclusion criteria for the *TP53*-wild-type (*TP53*-wt) group were the lack of any cytogenetic anomaly. Characteristics of patients at diagnosis are summarized in Table 2. Patients were stratified into major cytogenetic categories, based on NCCN CLL Guidelines^28^: favorable (del13q as a sole aberration), neutral (normal karyotype, trisomy 12), and unfavorable (11q and/or 17p deletion). Peripheral blood mononuclear cell (PBMC) samples from 7 of 26 untreated CLL patients (4 with *TP53*-wt and 3 with *TP53*-mut) were collected and cryopreserved at the Hematology Unit, Azienda Ospedaliera Universitaria Integrata (AOUI) in Verona (Italy) on approval from the local Ethics Committee (Comitato Etico per la Sperimentazione, AOUI). In accordance with the Declaration of Helsinki, all patients and donors provided written informed consent for the collection and use of their blood samples and clinical annotation for research purposes.

**Table 2:** Clinical and biological features of CLL patients

	*TP53*-wt (*n* = 16)	*TP53*-mut (*n* = 10)
	Total, *n*	Total, *n*
Age at the diagnosis		
median (range), years	63 (43–81)	70 (52–85)
Gender		
male	9 (56%)	6 (60%)
female	7 (44%)	4 (40%)
Stage		
Binet A	16 (100%)	9 (90%)
Binet B	0 (−)	1 (10%)
Binet C	0 (−)	0 (−)
IGHV^a^		
Mutated	16 (100%)	2 (20%)
Unmutated	0 (−)	7 (70%)
NA	–	1 (10%)
CD38^b^		
Negative	16 (100%)	4 (40%)
Positive	0 (-)	6 (60%)
Cytogenetics		
Favorable	0 (-)	2 (20%)
Neutral	16 (100%)	3 (30%)
Unfavorable	0 (−)	3 (30%)
NA	–	2 (20%)
Follow-up		
Median (range), years	4 (1–6)	5 (2–19)
Disease progression, requiring treatment during follow-up	2 (13%)	7 (70%)
TTFT		
Median (range), months	14 (7–22)	49 (3–212)

*NA* not available

^a^*IGHV* sequencing utilized a 2% cutoff to discriminate mutated from unmutated *IGHV*

^b^CD38 was determined using a 30% cutoff

### CLL cell preparation

PBMCs were isolated by Ficoll-hypaque centrifugation (Lymphoprep, Nicomed, Oslo, Norway) and stored in liquid nitrogen. Upon thawing, only samples with at least 85% viability, assessed using 7-amino-actinomycin dye (BD Biosciences, San Jose, CA, USA) and flow cytometry (FACSCanto II, Becton Dickinson), were processed further. CLL cells from samples with < 70% B cells were isolated by negative selection using Human B-Cell Enrichment Kit (Stem Cell Technologies, Vancouver, Canada). After separation, cell purity was routinely above 98%, as assessed with CD19 staining and flow cytometry (FACSCanto II, Becton Dickinson).

### Genetic analysis

Cytogenetic abnormalities were evaluated by cytogenetic and fluorescence in situ hybridization (FISH), according to the hierarchical risk model of FISH anomalies.^29^ The mutation hot-spots of the *TP53* (exons 4–9, including splicing sites; RefSeq NM_000546.5) gene were analyzed by PCR amplification and direct sequencing of high molecular weight genomic DNA, as previously described.^30^

### Reduced thiols

Levels of reduced thiols were detected by ThiolTracker Violet (Thermo Fisher Scientific), according to the manufacturer’s instruction. In brief, final 5 μM ThiolTracker was added to cell suspension and incubated at 37 °C for 30 minutes. After incubation, cells were stained with anti-CD19-APC and anti-CD5-PECy7 (BD Biosciences) for 15 minutes and analyzed by flow cytometry (FACSCanto II, Becton Dickinson). Analysis was gated on CLL cells, identified as CD19/CD5 co-expressing cells.

### Statistical analysis

Analysis of variance analysis with GraphPad Prism 5 software or two-tailed *t* test were used to calculate *P* values. Statistically significant results were referred with a *P* value < 0.05. Values are the means of three independent experiments ( ± SD). In CLL patients, TTFT was calculated from the date of diagnosis to the date of initial therapy.^27^ Patients who did not receive any treatment during follow-up were censored at their last follow-up date. TTFT curves estimated using the Kaplan–Meier method were compared using the log-rank (Mantel–Cox) test.

## Results

### Mutant p53 increases mitochondrial ROS production

**Fig. 1:**
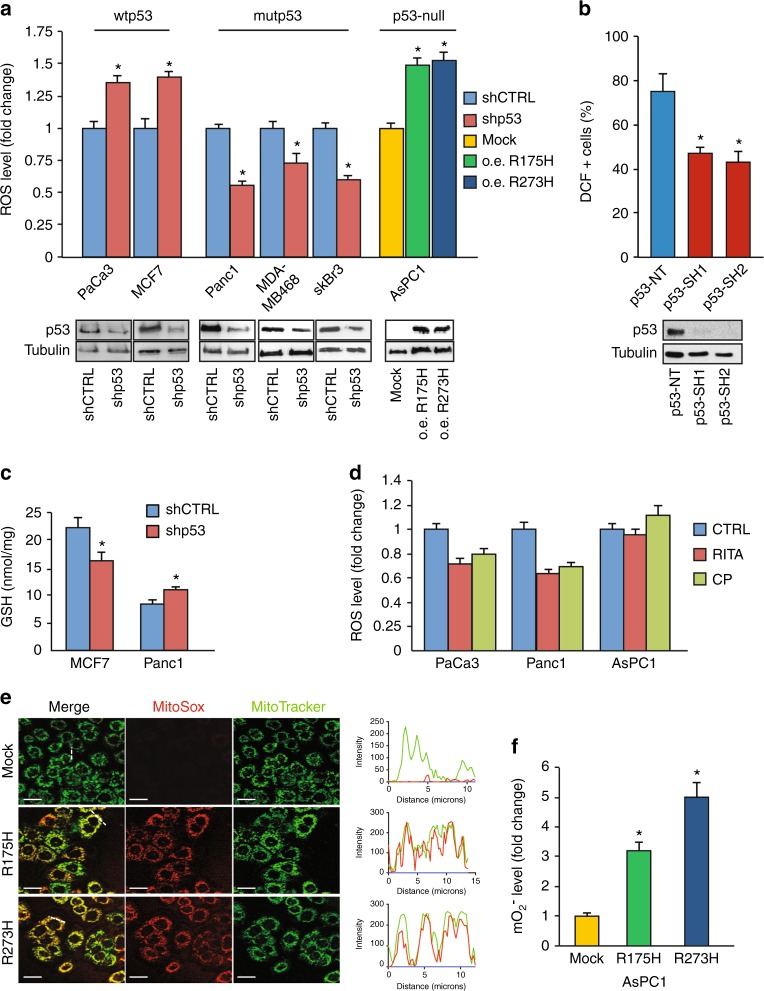
Mutant p53 proteins enhance ROS production in cancer cells. **a** The indicated cell lines were seeded in 96-well plates, incubated overnight, and transfected with the pRSuper-p53 vector and with plasmids for overexpression of mutant p53 (o.e. R175H; o.e. R273H), or their relative negative mock control (CTRL). DCF fluorescence intensity was analyzed by a multimode plate reader. Western blot of p53 was performed to test the effective knockdown of WT or mutant p53 and the overexpression of mutant p53 in the various cell lines indicated. **b** MDA-MB-231 cells stably silenced for p53 by lentiviral transduction with two different short hairpin sequences, were treated for 15 min with the ROS probe CM-H_2_DCFDA and then analyzed by FACS. Bars represent average (± SEM) of the percentage of DCF positive (+) cells measured by FACS from two independent biological experiments performed in technical duplicate. Statistically significant differences are indicated: *p < 0.05, ** *p* < 0.001. Western blot of p53 was performed to test the effective knockdown of mutant p53. **c** The intracellular oxidized and reduced glutathione were determined in MCF7 and Panc1 mutR273H-p53 cell lines after transient transfection with the pRsuper-p53 vector. **d** The indicated cell lines were seeded in 96-well plates, incubated overnight, and treated with 20 µM CP-31398 or 40 µM RITA for 48 h. DCF fluorescence intensity was measured by a multimode plate reader. **e** Live cell imaging: 48 h after transfection with plasmid coding for R175H or R273H mutant p53, or with the mock vector (CTRL), cells were incubated for 30 min with MitoSox probe (red) and Mitotracker Green (green). The RGB profile plotted along the dashed line drawn in the merge image is also shown. Merge and single channel images come from a single z-plane. Scale bar 10 μm. **f** Mitochondrial superoxide evaluation by MitoSox Red probe was analyzed with a multimode plate reader. Cells were seeded in 96-well plates, incubated overnight, and transfected with plasmid coding for R175H or R273H mutant p53, or the mock vector. All the experiments presented in this figure are representative of three biological replicates. *P* values were calculated with two-tailed *t* test. Statistical analysis: *p < 0.05

To study the functional role of GOF mutant p53 proteins in the regulation of ROS production, we first analyzed the endogenous level of ROS by staining diverse cancer cell lines with the DCF probe. Cancer cells with different missense mutations of the *TP53* gene (Table 1) had the endogenous level of ROS significantly higher than cells with wild-type *TP53* alleles in both pancreatic and breast cancer cell lines (Supplementary Figure 1), suggesting a possible involvement of mutant p53 in the production of ROS. When PaCa3 and MCF7 cell lines (expressing wtp53 protein) were knocked down for p53 expression by using liposome-mediated transient transfection assay, the ROS level increased, accordingly with the well-described antioxidant role of wild-type p53 (Fig. 1a). Conversely, the ROS level was significantly decreased after knockdown of GOF mutant p53 in Panc1, MDA-MB-468, and SkBr3 cancer cell lines (Fig. 1a). Consistent with this, exogenous overexpression of R175H or R273H mutant p53 proteins in AsPC1 cells (null for p53 expression) produced a drastic increase of ROS level (Fig. 1a). We further strengthened these data through lentivirus-mediated transduction and DCF fluorescence analysis by flow cytometry using two different sequences to knockdown mutant p53 expression (p53-SH1 or p53-SH2) or their negative control (p53-NT) in breast cancer MDA-MB-231 cells (Fig. 1b). Altogether these data finally confirm the pro-oxidant role of mutant p53 isoforms in pancreas and breast cancer cell lines. We also observed that intracellular GSH concentration increased after mutant p53 gene silencing, whereas it decreased after wild-type p53 downregulation (Fig. 1c), further supporting the pro-oxidant role of mutant proteins. These data highlight the opposite role of the tumor suppressor wild-type p53 and mutant p53 isoforms on the regulation of the intracellular redox status of cancer cells.

In the last few years, screening approaches have led to the identification of small molecules, such as CP-31398 and RITA, which can re-establish the wild-type transcriptionally competent conformation of mutant p53 proteins. These molecules are able to activate the wild-type-like p53 function in cancer cells expressing mutant proteins, triggering p53-dependent tumor suppression.^31,32^ Figure 1d shows that treatment with the p53-reactivators CP-31398 or RITA decreased ROS level in both cancer cells having wild-type p53 (PaCa3) and mutant p53 (Panc1); whereas ROS level in p53-null AsPC1 cells was unaffected by these compounds, suggesting that p53 expression is required for their action. These results confirm the antioxidant role of wild-type p53 and further indicate that the mutp53-depedent pro-oxidant function can be reverted by activating the wild-type-like conformation of the protein.

To further investigate the subcellular source of ROS production by mutant p53 we analyzed mitochondrial superoxide ions (O_2_ˉ·) by using MitoSox Red probe. We markedly observed fluorescence emitted by MitoSox Red probe after exogenous overexpression of R175H or R273H mutant p53 isoforms in p53-null AsPC1 cells (Figs. 1e, f) and also a strong colocalization of the fluorescence signals by MitoSox Red probe (revealing mitochondrial superoxide ions) and by MitoTracker Green (staining mitochondria) (Fig. 1e). These results indicate that mitochondria are a crucial source of ROS production induced by mutant p53 isoforms.

### The induction of ROS is functionally involved in the oncogenic effects of mutant p53

**Fig. 2:**
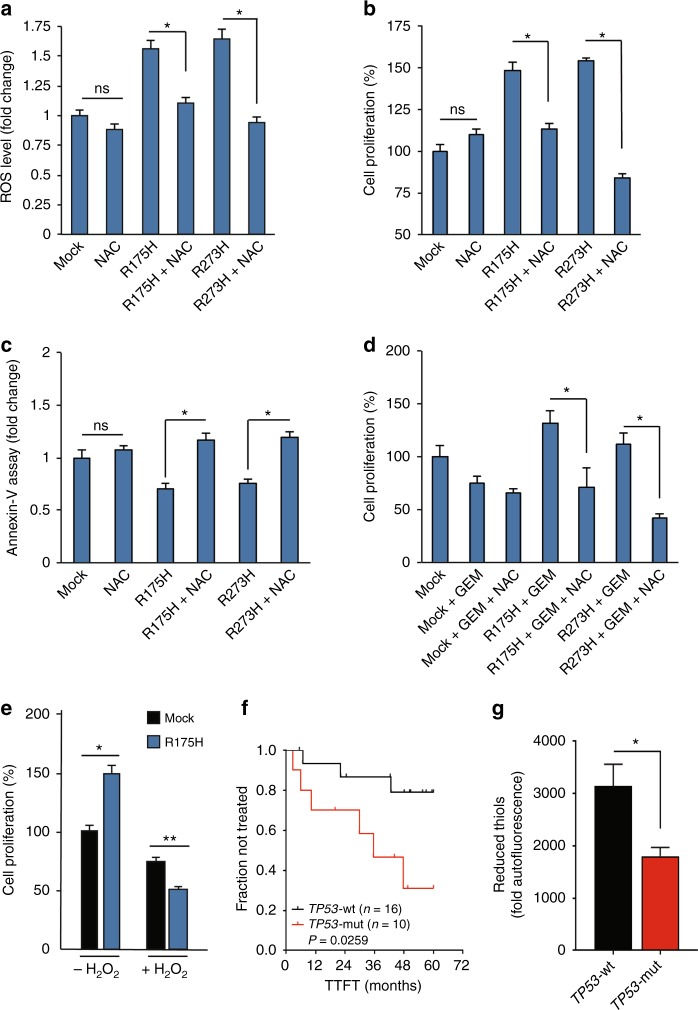
ROS induced by mutant p53 proteins are critical to mediate their oncogenic proprieties. **a**, b, **c** AsPC1-p53 null cells were transfected with R175H or R273H vector, or mock control, and concomitantly treated with 7 mM NAC for 24 h. **a** Intracellular ROS level was evaluated analyzing DCF fluorescence intensity using a multimode plate reader. **b** Cell proliferation was measured by Crystal Violet assay and **c** apoptosis was determined by the annexin V/FITC binding assay. Statistical analysis: *p < 0.05; *P* values were calculated with two-tailed *t* test. R175H vs R175H + NAC and R273H vs R273H + NAC. **d** AsPC1-p53 null cells were transfected with R175H and R273H vector, or negative control, and concomitantly treated with 7 mM NAC and 1 µM GEM for 24 h. Cell proliferation was measured by Crystal Violet assay. Statistical analysis: *p < 0.05; *P* values were calculated with two-tailed *t* test. R175H + GEM vs R175H + NAC + GEM and R273H + GEM vs R273H + NAC + GEM. **e** AsPC1-p53 null cells were transfected with mock vector or with vector to express R175H mutant p53. After 24 h cells were treated with 100 µM H_2_O_2_ for further 24 h. Cell proliferation was measured by Crystal Violet assay. Statistical analysis: *p < 0.05 or ** *p* < 0.01 *P* value were calculated with two-tailed *t* test. R175H vs mock. **f** Kaplan–Meier curves of TTFT for CLL patient subgroups defined by mutational status of *TP53* gene: wild-type (TP53-wt; *n* = 16) or mutated (TP53-mut; *n* = 10). **g** Comparison of reduced-thiol levels (detected by ThiolTracker probe) between CLL patients harboring *TP53* gene wild-type (TP53-wt; *n* = 4) or mutated (TP53-mut; *n* = 3). Statistical analysis was performed using the Student’s *t* test. Data are expressed as mean ± SEM. *: *P* < 0.05

To explore the role of ROS stimulation on the oncogenic effects of mutant p53 isoforms in cancer cells we analyzed cell proliferation, apoptosis, and response to GEM after addition of the radical scavenger NAC. First, we demonstrated that this antioxidant molecule was able to successfully counteract ROS production by overexpression of R175H or R273H mutant p53 isoforms in p53-null cells (Fig. 2a). The hyper-proliferative effect induced by mutant p53 was also completely reversed by NAC treatment (Fig. 2b). Furthermore, we demonstrated that the antiapoptotic effect induced by overexpression of mutant p53 isoforms was completely counteracted by NAC (Fig. 2c), demonstrating the functional involvement of ROS on these oncogenic events. As we previously published that mutant p53 conferred chemoresistence to GEM treatment in pancreatic cancer cells,^10^ we investigated whether this function may be mediated by ROS. Figure 2d shows that chemoresistance, observed after GEM treatment in the presence of mutant p53 overexpression, as compared with gemcitabine and mock conditions, was totally counteracted by the addition of NAC. By a therapeutic point of view, it is known that increasing ROS beyond a threshold level can inhibit cell proliferation inducing cell death, so we investigated whether cancer cells expressing mutant p53 may acquire sensitivity to pro-oxidant agents. As shown in Fig. 2e, expression of R273H mutant p53 enhances cancer cell sensitivity to hydrogen peroxide, reversing the hyper-proliferative effect induced by mutant p53 and suggesting oxidant therapeutics in the treatment of cancer cells bearing mutant *TP53* gene. Concerning the clinical relevance of these data, we hypothesized that the presence of mutant *TP53* gene may have an impact on both the clinical outcome of CLL patients and in their oxidant status. Importantly, all CLL samples were collected at the time of diagnosis and therefore no treatment was performed on the CLL patients analyzed. As expected, we observed that *TP53* gene mutation was associated with a faster clinical progression in CLL (*P* = 0.0259), as shown by a shorter TTFT in Kaplan–Meier curves (Fig. 2f). Remarkably, leukemic patients harboring mutant *TP53* gene display also lower levels of reduced thiols (*P* = 0.0481), the major non-enzymatic antioxidant scavenger (Fig. 2g). Moreover, we sub-classified the CLL samples based on the p53 mutation types reported in Supplementary Table 2 and we analyzed TTFT of different patients’ subgroups. We observed a significant acceleration of the clinical progression in frameshift mutant *TP53* gene CLL patients as compared with WTp53 CLL, while missense mutants show a marked trend of faster clinical progression as compared with WTp53 CLL (Supplementary Figure 2). We also observed a significant acceleration of the clinical progression in exon 5–6 mutant *TP53* gene CLL patients as compared with WTp53 CLL, whereas exon 7 mutants show a strong trend of faster clinical progression as compared with WTp53 CLL (Supplementary Figure 2). Overall, all subgroups tested of CLL patients bearing mutant *TP53* gene show a worsening of the clinical outcome as compared with WTp53 CLL patients. These data might be further confirmed with a larger cohort of CLL patients with mutations in the *TP53* gene.

### Mutant p53 downregulates UCP2 expression through the inhibition of PGC-1α

**Fig. 3:**
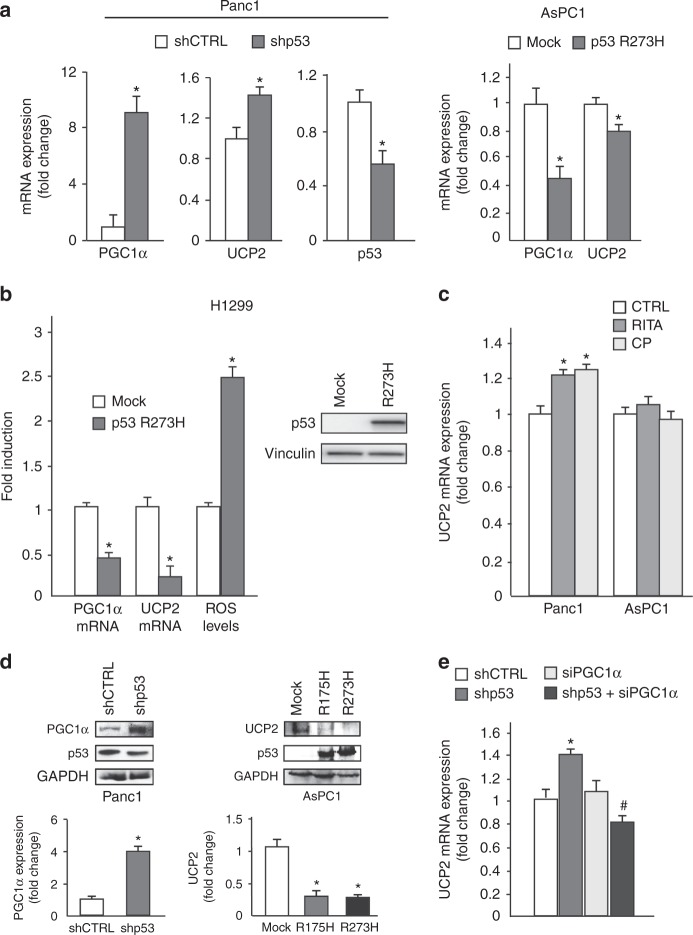
Mutant p53 downregulates UCP2 and PGC-1-α. **a** Panc1 mutR273H-p53 and AsPC1-p53 null cells were transfected with pRSuper-p53 vector and with plasmids for the ectopic expression of mutant p53-R273H or its relative negative control (CTRL). Gene expression analysis of the p53, UCP2, and PGC-1α was performed by RT-qPCR and was normalized to GAPDH mRNA. *p < 0.05. **b** H1299 p53-null cells stably expressing p53-R273H (clone H1) and respective mock control (clone C9) were used to analyze PGC-1α and UCP2 expression by RT-qPCR, normalized to GAPDH mRNA, and ROS levels with DCF probe. The western blotting was performed using 50 μg of whole-cell extracts and probed with p53 and vinculin antibodies *p < 0.05. **c** Panc1 mutR273H-p53 and AsPC1-p53 null cells were treated with 40 µM RITA and 20 µM CP-31398 for 48 h and gene expression analysis of UCP2 was performed by RT-qPCR and normalized to GAPDH mRNA. *p < 0.05. **d** Western blotting analysis of Panc1 mutR273H-p53 and AsPC1-p53 null cells transfected with the indicate plasmids. Western blotting was performed using 50 μg of whole-cell extracts, probed with the indicated antibodies and quantified with ImageJ software. **e** Panc1 mutR273H-p53 cells were transfected with pRSuper-p53 vector and siRNA-PGC-1α and relative controls and gene expression analysis of UCP2 was performed by RT-qPCR and normalized to GAPDH mRNA. *p < 0.05 shCTRL vs shp53; ^#^*p* < 0.05 shp53 vs shp53 + siPGC-1α. The experiments are representative of three biological replicates

As mitochondrial uncoupling protein UCP2 represents the first mechanism able to counteract the electron leakage from the respiratory chain and, in turn, to decrease the superoxide ions production in mitochondria, we investigated whether mutant p53 can modulate UCP2 expression and its transcriptional activator, peroxisome PGC-1α. Indeed, UCP2 expression is intimately linked to the activity of PGC-1α^33^ and PGC-1α/UCP2 axis is recognized as a relevant player in the maintenance of the mitochondrial redox balance.^19,34^ Herein, we observed that the depletion of endogenous mutant p53 in Panc1 cells determined the induction of both PGC-1α and UCP2 mRNAs, whereas the exogenous expression of the R273H mutp53 isoform inhibited their expression in p53-null AsPC1 (Fig. 3a). We further confirmed the mutp53-dependent stimulation of ROS associated to the downregulation of PGC-1α and UCP2 mRNAs in a clone of lung carcinoma H1299 cells stably expressing R273H mutp53 (Fig. 3b).

In accordance with the repressive effect of mutant p53 on the PGC-1α/UCP2 axis, the p53-reactivators CP-31398 or RITA increased the expression level of UCP2 mRNA in mutant p53 Panc1 cells, whereas failed to modulate UCP2 expression in p53-null AsPC1 cells, used as negative control (Fig. 3c). We also demonstrated at the protein level the inhibitory role of mutant p53 on the expression of PGC-1α and UCP2 in Panc1 and AsPC1 cells (Fig. 3d). Notably, Fig. 3e shows that the stimulation of UCP2 mRNA by mutp53 knockdown was completely counteracted by co-transfecting Panc1 cells with siRNA-PGC-1α, demonstrating the functional involvement of PGC-1α inhibition in the repression of UCP2 mRNA by mutant p53.

### UCP2 inhibition by mutant p53 increases ΔΨm without damaging mtDNA

**Fig. 4:**
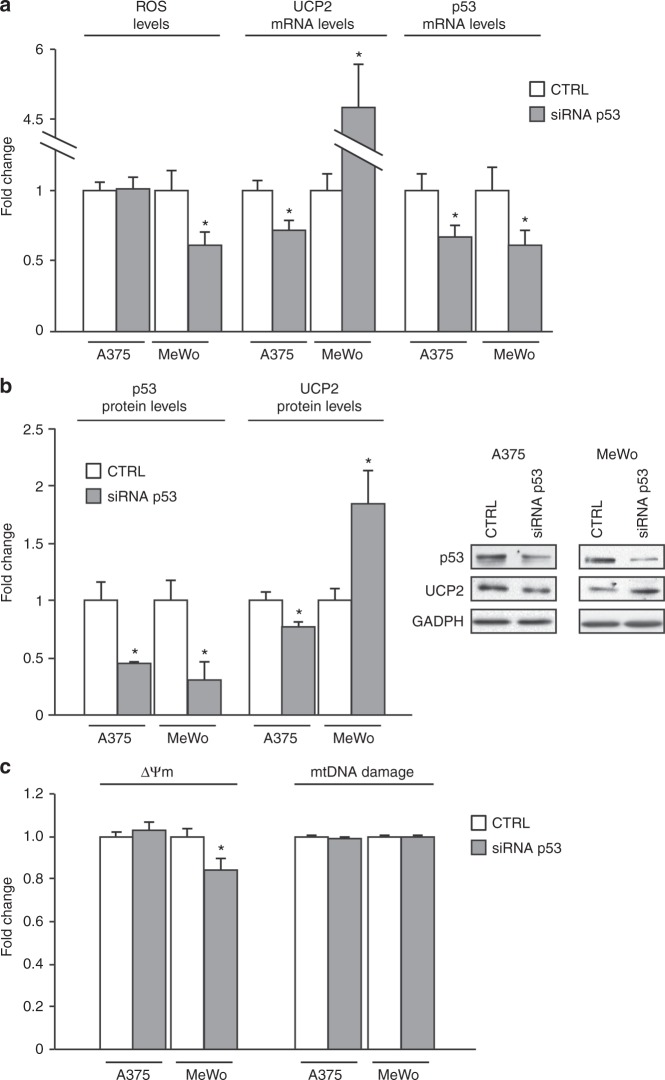
Mutant p53 increases ΔΨm without damaging mtDNA. **a** Melanoma cell lines A375 (WTp53) and MeWo (mutant p53) were transfected with control or siRNA-p53. H_2_O_2_ production was evaluated with the Amplex Red Kit, whereas UCP2 and p53 levels were determined by RT-qPCR. *p < 0.05. **b** Protein expression analysis of p53 and UCP2 was performed by western blotting and normalized to GAPDH expression. *p < 0.05. **c** Mitochondrial membrane potential (ΔΨm) was determined by TMRM fluorescencent probe, and mtDNA damage was evaluated with a qPCR approach as described in Material and Methods section. *p < 0.05 CTRL vs siRNA-p53. The experiments are representative of three biological replicates

To confirm these observations also in other tumor types we downregulated p53 expression in both A375 melanoma cells, bearing wild-type p53, and MeWo melanoma cells, bearing E258K mutant p53, and analyzed ROS and UCP2 levels. Figure 4a shows that mutant p53 silencing decreased H_2_O_2_ production and increased UCP2 mRNA expression, whereas wild-type p53 silencing did not affect H_2_O_2_ production and only slightly decreased UCP2 mRNA. At the protein level, we obtained similar results (Fig. 4b), confirming also in melanoma cells that the UCP2-mediated pro-oxidant effect is a phenomenon acquired by mutant p53 isoforms. Furthermore, we analyzed the ΔΨm and mtDNA damage after knockdown of p53 expression in both cell lines. Figure 4c shows that ΔΨm decreased after mutant p53 silencing, whereas there were no changes in mtDNA damage. Overall these data suggest that mutp53-dependent UCP2 inhibition determined mitochondrial superoxide production and hyperpolarization without damaging mtDNA.

### Mutant p53-dependent downregulation of the PGC-1α/UCP2 axis is mediated by the blockage of SESN1/AMPK signaling

**Fig. 5:**
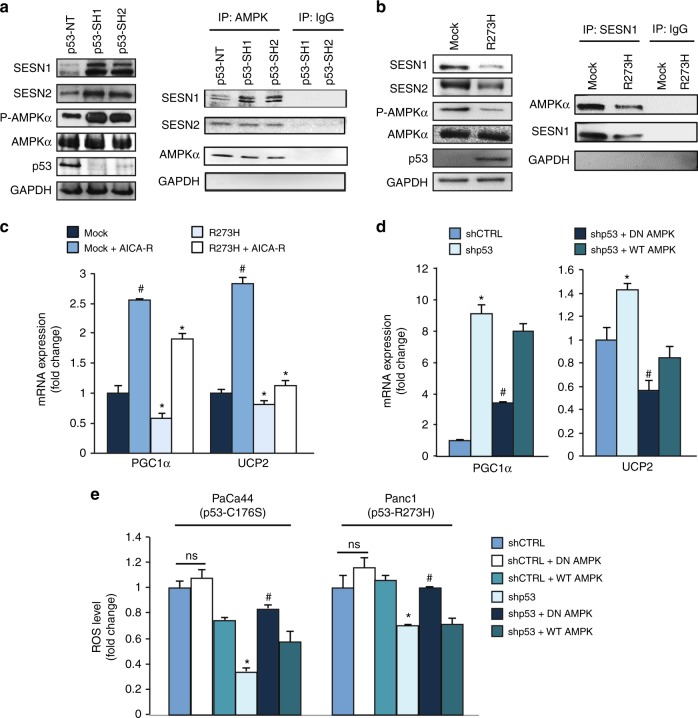
Mutant p53 inhibits UCP2 and PGC-1α through the inhibition of SESN1/AMPK signaling. **a** MDA-MB-231 cells were transduced with lentiviruses containing p53-SH1 or p53-SH2 vectors for mutant p53 silencing or their non-targeting negative control (NT). Left panel: western blotting was performed using 50 μg of whole-protein extracts and probed with the indicated antibodies. The p53 expression was shown as control of p53 knockdown efficacy and the GAPDH expression was used as control of equal proteins loading. Right panel: AMPK was immunoprecipitated from protein extracts using anti-rabbit AMPK antibody (IP: AMPK) and western blot analysis was performed using indicated antibodies. Protein extracts from cells silenced for p53 expression with p53-SH1 or p53-SH2 vectors were also immunoprecipitated with rabbit IgG as control. The blot exhibits equivalent AMPK levels in all samples. **b** H1299 p53-null cells stably expressing R273H mutant p53 (clone H1) and its respective mock control (clone C9) were used to confirm the regulation of SESN1:AMPK by mutant p53. Left panel: western blotting was performed using 50 μg of whole-protein extracts and probed with the indicated antibodies. Right panel: SESN1 was immunoprecipitated from protein extracts using anti-SESN1 antibody (IP: SESN1) and western blot analysis was performed using indicated antibodies. Protein extracts were also immunoprecipitated with IgG as control. **c** AsPC1-p53 null cells were transfected with the vectors for the ectopic expression of p53-R273H and its mock control and treated with 1 mM AICA-R for 48 h. Gene expression analysis of the UCP2 and PGC-1α was performed by RT-qPCR and normalized to GAPDH mRNA. ^#^*p* < 0.05 mock vs mock + AICA-R; *p < 0.05 mock vs R273H and R273H vs R273H + AICA-R. **d** Panc1 mutR273H-p53 cells were transfected for 48 h with the indicated vectors and their relative negative controls. Gene expression analysis of UCP2 and PGC-1α was performed by RT-qPCR and normalized to GAPDH mRNA. *p < 0.05 shp53 vs CTRL; ^#^*p* < 0.05 shp53 + DN-AMPK vs shp53 or shp53 + WT-AMPK. **e** The indicated cell lines were transfected with pRSuper-p53, DN-AMPK, WT-AMPK vectors, or negative controls. ROS levels were analyzed using DCF probe by a multimode plate reader. *p < 0.05 shp53 vs CTRL; ^#^*p* < 0.05 shp53 + DN-AMPK vs shp53 or shp53 + WT-AMPK. The experiments are representative of three biological replicates

As AMPK signaling pathway has a crucial role in many biological functions, including the induction of PGC-1α, we examined whether mutant p53 might inhibit PGC-1α/UCP2 axis through the upstream blockage of AMPK. We demonstrate that R273H mutant p53 inhibited the level of SESN1 and SESN2 mRNAs (Supplementary Figure 3) and that knockdown of R280K mutant p53 increased the protein expression of SESN1 and SESN2, as well as the phospho tyrosine-172 AMPK level without affecting the total amount of AMPK expression (Fig. 5a), as previously observed also for R175H and R273H mutant p53 isoforms,^15^ suggesting that the inhibition of SESNs and of AMPK signaling is a common event triggered by various hotspot mutant isoforms of p53. Importantly, we demonstrated by immunoprecipitation that SESN1-AMPK binding increased in R280K mutant p53 silencing conditions, likely as a consequence of SESN1 increase (Fig. 5a). This result is in accordance with the enhanced level of P-AMPK as SESN1:AMPK complex favors AMPK phosphorylation by upstream kinases.^35^ Figure 5a also shows that the formation of SESN2:AMPK complex was not regulated by mutant p53, despite mutant p53 inhibited the expression of SESN2, further improving the findings about SESN1:AMPK complex. To confirm the results shown in Fig. 5a, we used a clone of p53-null H1299 cells stably expressing R273H mutant p53 and its mock control clone. Figure 5b shows that expression of R273H mutant p53 decreased the protein expression of SESN1 and SESN2, as well as the P-AMPK level without affecting the total amount of AMPK expression. We also demonstrated by reciprocal SESN1 immunoprecipitation assay that R273H mutant p53 decreased SESN1 expression and, consequently, SESN1 binding to AMPK.

To investigate the functional role of AMPK signaling in the inhibition of PGC-1α/UCP2 axis by mutant p53, we treated cells with AICA-R, a chemical activator of AMPK (Supplementary Figure 4). Figure 5c shows that the repression of both PGC-1α and UCP2 mRNAs by overexpression of mutant p53 was reverted by AICA-R. In addition, we further demonstrated that transfection with a DN isoform of AMPK γ, as compared with transfection with wild-type AMPK isoform γ (WT-AMPK), was able to significantly counteract the induction of both PGC-1α and UCP2 mRNAs after silencing of endogenous mutant p53 (Fig. 5d). Altogether these data indicate that mutp53-dependent downregulation of the PGC-1α/UCP2 axis is mediated by the blockage of AMPK signaling.

In order to investigate whether AMPK also plays a role in the final pro-oxidant effect of mutant p53, we analyzed ROS level after silencing endogenous mutant p53 with the concomitant transfection of DN-AMPK or WT-AMPK. Figure 5e shows that ROS level decreased in mutp53-knockdown conditions and it was recovered by DN-AMPK. Altogether these data indicate that the pro-oxidant inhibition of the PGC-1α/UCP2 axis by mutant p53 is functionally owing to the AMPK signaling inhibition, which might be, at least partially, ascribed to the inhibition of SESN1 expression.

### UCP2 blockage has a major role on the pro-oxidant and oncogenic effect of mutant p53

**Fig. 6:**
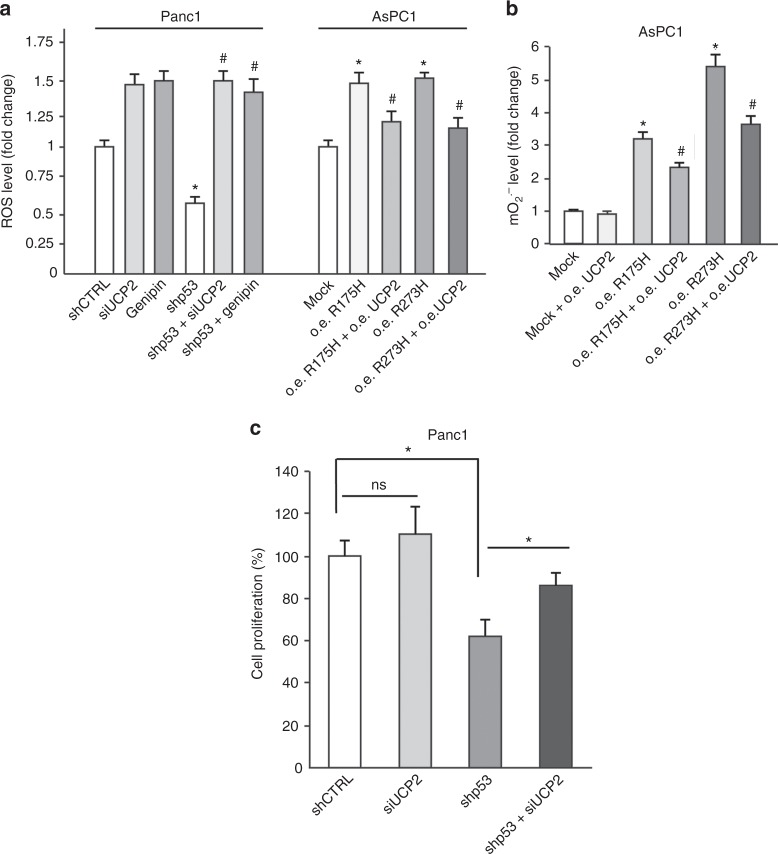
Mitochondrial superoxide production is due to mutp53-dependent UCP2 inhibition. **a** Panc1 mutR273H-p53 and AsPC1-p53 null cells were transfected with pRSuper-p53 and vector for mutant p53 ectopic expression, respectively, and their relative controls. In addition, Panc1 cells were co-transfected with siRNA-UCP2, or treated with 150 μM genipin for 24 h; while AsPC1 cells were co-transfected with the UCP2 vector. ROS production, corresponding to DCF fluorescence intensity, was analyzed by a multimode plate reader. *p < 0.05 shCTRL vs shp53 or mock vs R175H or R273H; ^#^*p* < 0.05 shp53 vs shp53 + siUCP2 or shp53 + genipin (Panc1 cells); R175H or R273H vs R175H + UCP2 or R273H + UCP2 (AsPC1 cells). **b** AsPC1 cells were transfected with the vectors for UCP2 and/or mutant p53 expression. Mitochondrial superoxide production was determined with the MitoSox Red probe by a multimode plate reader. *p < 0.05 CTRL vs R175H or R273H; ^#^*p* < 0.05 R175H or R273H vs R175H + UCP2 or R273H + UCP2. **c** Panc1 cells were transfected for 48 h with shp53 and/or siUCP2 and their relative negative controls. Cell proliferation was measured by Crystal Violet assay. *p < 0.05 shCTRL vs shp53; shp53 vs shp53 + siUCP2. The experiments are representative of three biological replicates

To functionally demonstrate that the UCP2 blockage has a role on the pro-oxidant effect of mutant p53, we used the known UCP2 inhibitor genipin,^25^ or modulated UCP2 expression by siRNA or exogenous overexpression as checked in Supplementary Figure 5. In Fig. 6a we demonstrated that ROS level decreased after mutant p53 knockdown was recovered by siRNA-UCP2 or by genipin in Panc1 cells. In addition, ROS level increase by R273H or R175H mutant p53 overexpression in p53-null AsPC1 cells was counteracted by UCP2 overexpression (Fig. 6a). In accordance with the observation that mitochondria represent a main source of ROS by mutant p53 we observed similar results analyzing mitochondrial superoxide ions (Fig. 6b). To finally demonstrate that UCP2 inhibition is involved in the oncogenic hyper-proliferative effect of mutant p53 we analyzed cancer cell proliferation after the concomitant silencing of mutant p53 and UCP2. Figure 6c reveals that UCP2 knockdown was able to partially reverse the antiproliferative effect of mutant p53 blockage. Altogether these data indicate that UCP2 inhibition is a mechanism by which mutant p53 isoforms exert their oncogenic pro-oxidant functions.

### PGC-1α/UCP2 axis is not regulated by the endogenous basal level of wild-type p53

**Fig. 7:**
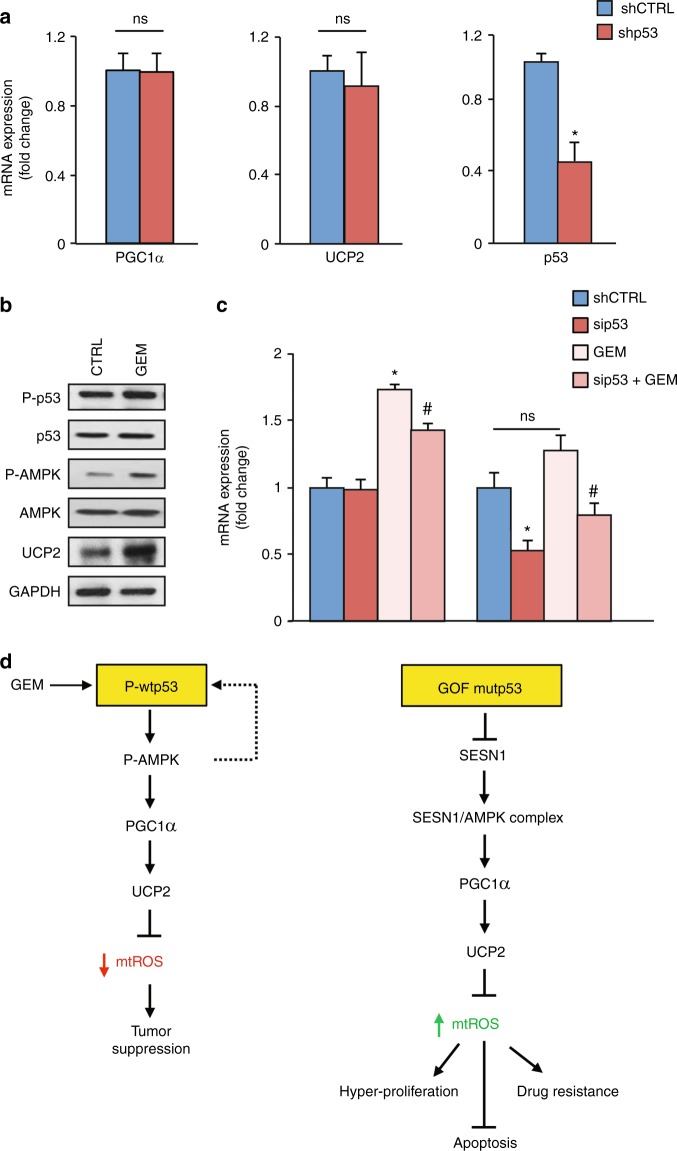
Gemcitabine triggers WTp53 and UCP2 expression. **a** PaCa3 cells (WTp53) were transfected for 48 h with the pRSuper-p53 vector or its relative negative control (shCTRL). Gene expression analysis of p53, UCP2, and PGC-1α was performed by RT-qPCR and normalized to GAPDH mRNA. Protein expression analysis of p53, PGC-1α, UCP2, and GAPDH was performed by western blotting. *p < 0.05. **b** Western blotting analysis of PaCa3 cells treated with 1 µM GEM for 24 h using 50 μg of whole-cell extracts and probed with the indicated antibodies. **c** PaCa3 cells were treated with 1 µM GEM for 24 h and/or transfected with the siRNA-p53 or its relative siRNA scramble (CTRL). Gene expression analysis of UCP2 and p53 was performed by RT-qPCR and normalized to GAPDH mRNA. *p < 0.05 CTRL vs GEM or sip53; ^#^*p* < 0.05 GEM vs sip53 + GEM. The experiments are representative of three biological replicates. **d** Model of the mechanisms identified in the present study

To confirm that PGC-1α/UCP2 inhibition is specifically a mutant p53-related GOF mechanism we examined whether the endogenous basal level of wild-type p53 is able to modulate this axis. Figure 7a shows that in PaCa3 cells wild-type p53 knockdown failed to modulate both mRNA and protein expression levels of PGC-1α, a crucial transcriptional activator of the UCP2 gene. In addition, wild-type p53 knockdown also failed to modulate UCP2 mRNA and protein expression levels (Fig. 7a), in accordance with the lack of PGC-1α regulation and the absence of p53 binding sequences in the regulatory regions of the UCP2 gene. As it has been described that mutant p53 can bind to wild-type p53 as heterodimers acting as dominant negative regulators of wtp53 functionality,^36^ we expressed the R175H mutp53 isoform in PaCa3 cells bearing wild-type *TP53* gene. Supplementary Figure 6 shows that R175H mutp53 increased ROS, in accordance with the suppression of the antioxidant role of wtp53, without altering the expression level of PGC-1α and UCP2. These data further confirm the statement that PGC-1α/UCP2 axis is not regulated by endogenous basal level of wtp53 and its inhibition is a mutp53-related GOF mechanism.

### Chemotherapy stimulates UCP2 through wtp53 activation

As wild-type p53 generally needs stimulation to become active, we triggered wild-type p53 phosphorylation by treating pancreatic cancer PaCa3 cells with the chemotherapeutic drug GEM. Figure 7b shows that GEM was able to induce P-p53, P-AMPK and UCP2, suggesting that, once activated, wild-type p53 might induce the antioxidant AMPK/UCP2 axis, in accordance with its antioxidant and tumor suppressive functions. To investigate the functional involvement of wild-type p53 in the UCP2 stimulation by GEM, we analyzed UCP2 and p53 mRNAs after p53 silencing and GEM cell treatment. Figure 7c shows that wild-type p53 knockdown decreased GEM-mediated induction of UCP2 mRNA. Altogether these data indicate that the AMPK-meditated pro-oxidant UCP2 inhibition is a mechanism linked to GOF mutant p53 and not to the endogenous basal level of wild-type p53, which on the contrary, upon activation, may promote the expression of the antioxidant UCP2 gene, likely through the AMPK signaling.

A schematic representation of the molecular mechanisms identified in this study is provided in Fig. 7d. Overall, it emerges that wild-type and mutant p53 have an opposite regulation of mitochondrial ROS and that this dual effect is based on the divergent modulation of the AMPK/PGC-1α/UCP2 axis.

## Discussion

ROS are persistently elevated in cancer cells as a consequence of increased metabolic activity, mitochondrial dysfunction, and activation of oncogenes.^37^ Some studies unveil that ROS play exceptional relevance in the development and progression of tumors being involved in the main features of aggressive cancer cell behavior, including genome instability, cellular hyper-proliferation, epithelial–mesenchymal transition, invasion, and metastasis.^38^ However, the role of ROS in cancer cell biology is highly contextual and dependent on the nature of the stress, tumor tissue, and stage.^39^ Indeed, despite they can stimulate tumorigenesis and cancer development, a severe increase in ROS level may induce cell death following a non-specific injury of macromolecules and cellular organelles.^40^

It is emerging that mutant p53 proteins, contrarily to their wild-type p53 counterpart, fail to exert antioxidant properties rather sustaining a controlled increase of intracellular ROS, resulting in cancer progression. In accordance with this statement, we recently demonstrated that mutant p53 inhibits the expression of sestrins,^15^ which are a family of highly conserved stress-inducible proteins and important negative regulators of both ROS and mTORC1 signaling pathways.^41^ The antioxidant effect of sestrins mainly occurs stimulating the p62-dependent autophagic degradation of the protein Keap1, an inhibitor of the crucial antioxidant transcription factor NRF2,^42^ and through its intrinsic peroxidase activity.^41^ In addition, sestrins can attenuate ROS accumulation by blocking mTORC1 activation. This inhibition of mTORC1 activity is achieved either via the AMPK-dependent phosphorylation and activation of TSC2 and consequent inhibition of the GTPase Rheb or via inhibition of the GTPase Rag and consequent prevention of the lysosomal localization of mTORC1 triggered by amino acids.^42^ Beyond the inhibition of sestrins, Rotter and colleagues elucidated that R273H mutant p53 interferes with the normal response of tumor cells to oxidative stress being able to attenuate the antioxidant function of NRF2.^43^ Furthermore, Boudreau et al.^44^ revealed that mutant p53 proteins, contrarily to the wild-type counterpart, enhance the expression of the NADPH oxidase NOX4, resulting in an increase of intracellular ROS levels, which sustain an invasive phenotype of breast cancer cells. Khromova et al. demonstrated that p53 hotspot mutants increase intracellular ROS level determining the augmentation of the number of vessels in HCT116 colon carcinoma xenografts, thus accelerating cancer growth. They revealed that the stimulation of tumor angiogenesis occurs through ROS upregulation causing activation of HIF1/VEGF-A pathway.^45^ More recently, some studies revealed that mutant p53 proteins suppress the expression of SLC7A11, a key component of the cystine/glutamate antiporter system xC^-^, diminishing glutathione synthesis and resulting in redox imbalance.^46,47^

In the present study, we demonstrate that various hotspot mutant p53 proteins determine mitochondrial O_2_ˉ· level increase in a number of cancer cell lines from different tissue origins, including pancreas, breast, skin, and lung. As we recently published that low expression of the antioxidant gene catalase confers redox hypersensitivity in CLL patients,^48^ we here investigated the involvement of mutant p53 in the redox regulation of CLL and we find a correlation between the poor clinical outcome of CLL patients having mutant *TP53* gene with a decreased amount of intracellular reduced thiols. Overall, we identify a novel mechanism by which mutant p53 proteins can stimulate their oncogenic pro-oxidant conditions through the inhibition of SESN1 expression and, consequently, of the SESN1:AMPK complex amount, resulting in the downregulation of the PGC-1α/UCP2 axis. AMPK is considered a master regulator of cellular metabolism and it has been shown to control the expression of several mitochondrial enzymes and proteins,^49^ including PGC-1α, which has been widely described to act as master regulator of mitochondrial biogenesis.^50^ Previous work has demonstrated that PGC-1α can function as a regulator of its own gene expression in a feedforward loop.^51^ More recently, Jager et al.^52^ demonstrated that AMPK directly phosphorylates PGC-1α at threonine-177 and serine-538 and that these phosphorylations are required for the PGC-1α-dependent induction of the PGC-1α promoter, indicating that AMPK phosphorylation of PGC-1α initiates many of the important gene regulatory functions of AMPK. Mechanistically, these AMPK-mediated phosphorylations might modulate the ability of PGC-1α to dock on certain transcription factors or affect the binding or function of other cofactors in the PGC-1α coactivator complex. PGC-1 family members of transcription coactivators can stimulate the expression of mitochondrial uncoupling proteins, as UCP2 and UCP3.^53^ In particular, UCP2 expression is intimately linked to the activity of the PGC-1α and in pancreatic beta-cells, PGC-1α has been shown to stimulate UCP2 gene expression via binding to two proximal regions of the UCP2 promoter.^33^

In addition to the antioxidant property of UCP2 by allowing the flux of protons from the intermembrane space to the mitochondrial matrix, the channel formed by uncoupling proteins can also promote the mitochondrial efflux toward the cytosol of pyruvate and of Krebs cycle intermediates, regulating glucose and glutamine oxidation.^54^ We have recently found that UCP2 also induces the expression of GLUT1 and pyruvate kinase isoform M2 sustaining the glycolytic phenotype of pancreatic adenocarcinoma cells.^55^ Therefore, the PGC-1α/UCP2 axis not only has mere antioxidant properties, but also has a central role in regulating the energetic metabolism of the cells. Then, our study also provides the molecular basis for future metabolic studies that might further clarify the alterations of cancer metabolism driven by mutant p53.

In conclusion, it is conceivable that GOF mutant p53 isoforms contribute to enhance ROS levels in cancer cells through a coordinated regulation of redox-related enzymes and signaling pathways, including SESN1/AMPK/PGC-1α/UCP2 axis, favoring cancer cell growth without damaging mitochondria. As ROS can produce genomic DNA damage,^56^ it is conceivable that ROS enhancement by mutant p53 may contribute to the induction of genomic DNA damage and genomic instability, which are typical hallmarks of cancer cells bearing mutant *TP53* gene.^8,57^ Intriguingly, from a therapeutic point of view, the intracellular ROS enhancement driven by mutant p53 might represent an “Achilles heel” of cancer cells carrying mutant *TP53* gene, as revealed by the mutp53-dependent acquisition of cell sensitivity to H_2_O_2_ treatment. These results suggest that cancer cells expressing mutant p53 proteins can be significantly more sensitive to pro-oxidant drugs, as compared with the wild-type counterpart, leading to overwhelming ROS accumulation and cancer cell death. This might provide new therapeutic opportunities to be further considered for clinical studies in cancer patients bearing mutant *TP53* gene.

## Electronic supplementary material

Supplementary Figure 1

Supplementary Figure 2

Supplementary Figure 3

Supplementary Figure 4

Supplementary Figure 5

Supplementary Figure 6

Legends to Supplementary Figures

Supplementary Table 1

Supplementary Table 2

## Availability of data and materials

Summary data supporting the results reported in this article can be requested by emailing the corresponding author.
