# Supplementary figures and images for: Mutant p53 blocks SESN1/AMPK/PGC-1α/UCP2 axis increasing mitochondrial O2ˉ· production in cancer cells
Source: Br J Cancer. 2018 Oct 15;119(8):994–1008. doi: 10.1038/s41416-018-0288-2 (PMC6203762; doi:10.1038/s41416-018-0288-2)

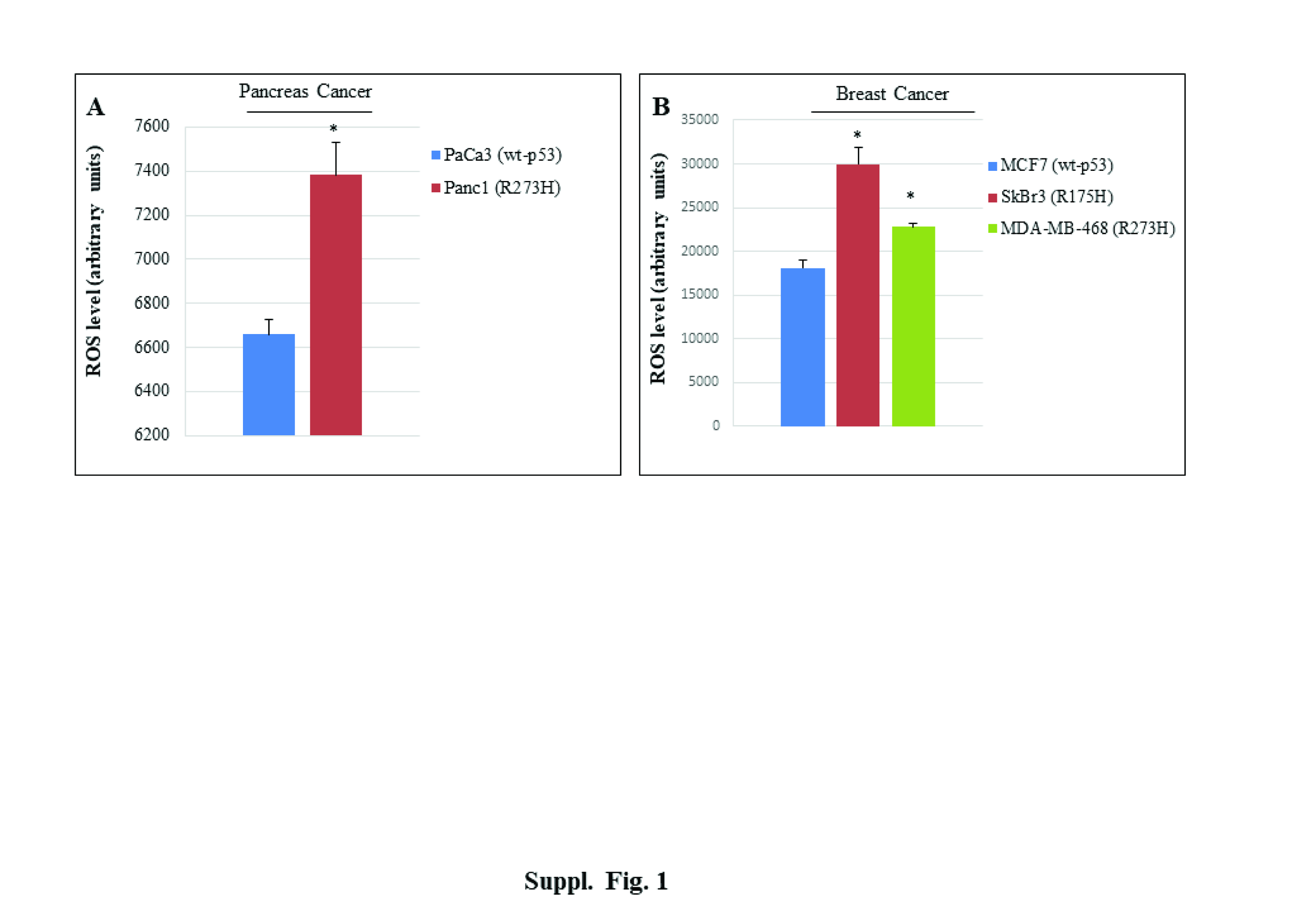

Supplement: Supplementary file 1 — Supplementary Figure 1 [file 41416_2018_288_MOESM1_ESM.tif]

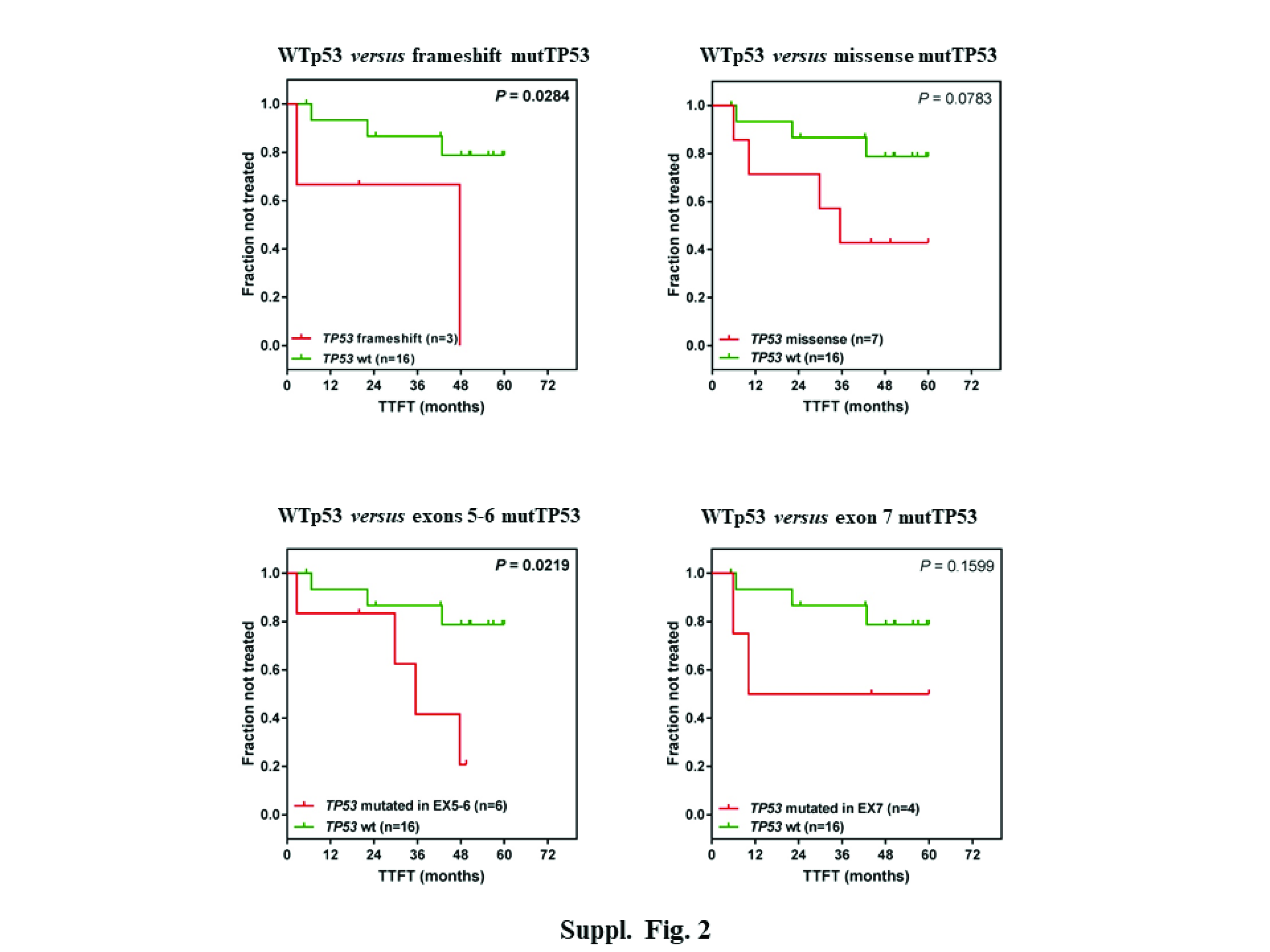

Supplement: Supplementary file 2 — Supplementary Figure 2 [file 41416_2018_288_MOESM2_ESM.tif]

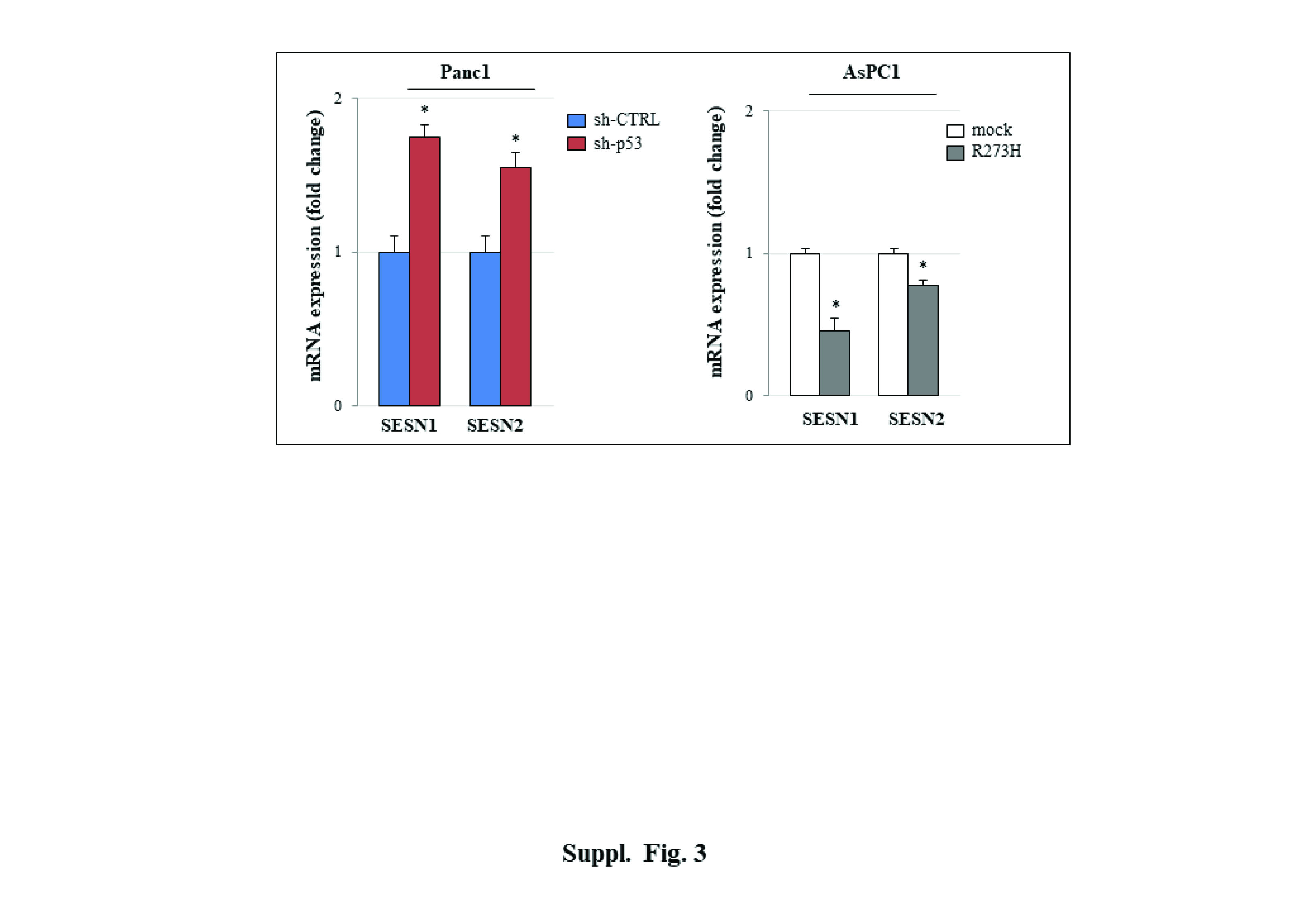

Supplement: Supplementary file 3 — Supplementary Figure 3 [file 41416_2018_288_MOESM3_ESM.tif]

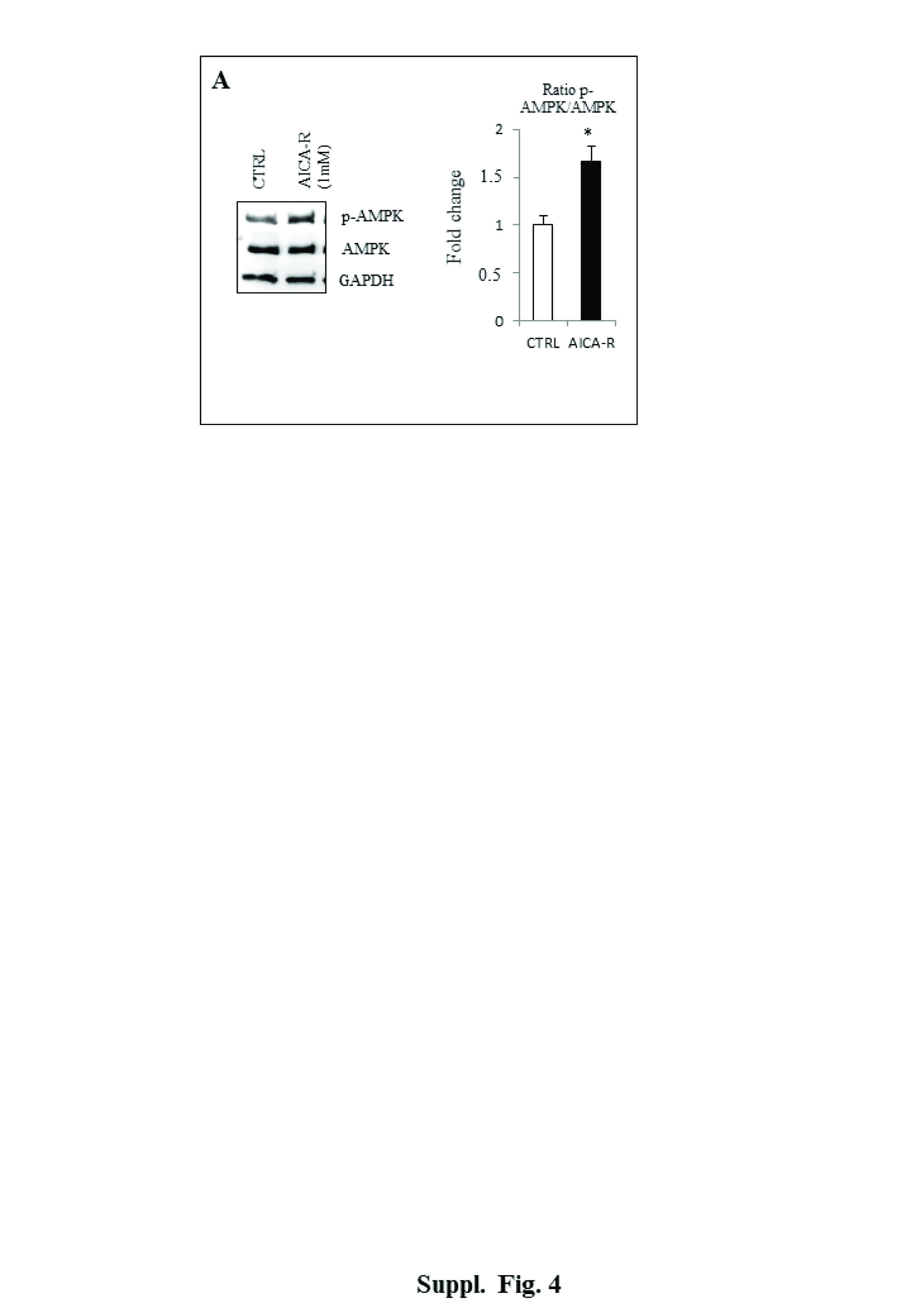

Supplement: Supplementary file 4 — Supplementary Figure 4 [file 41416_2018_288_MOESM4_ESM.tif]

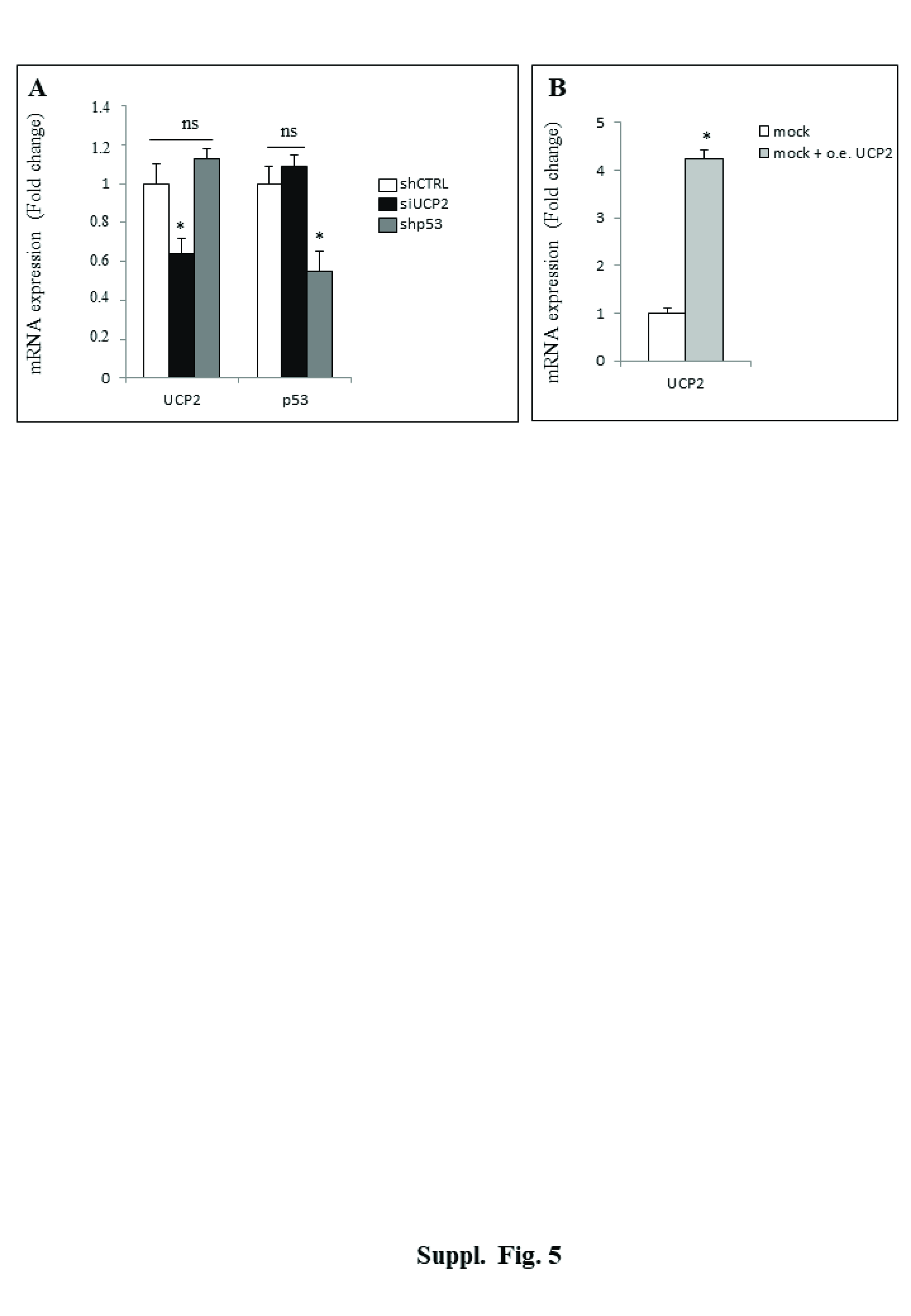

Supplement: Supplementary file 5 — Supplementary Figure 5 [file 41416_2018_288_MOESM5_ESM.tif]

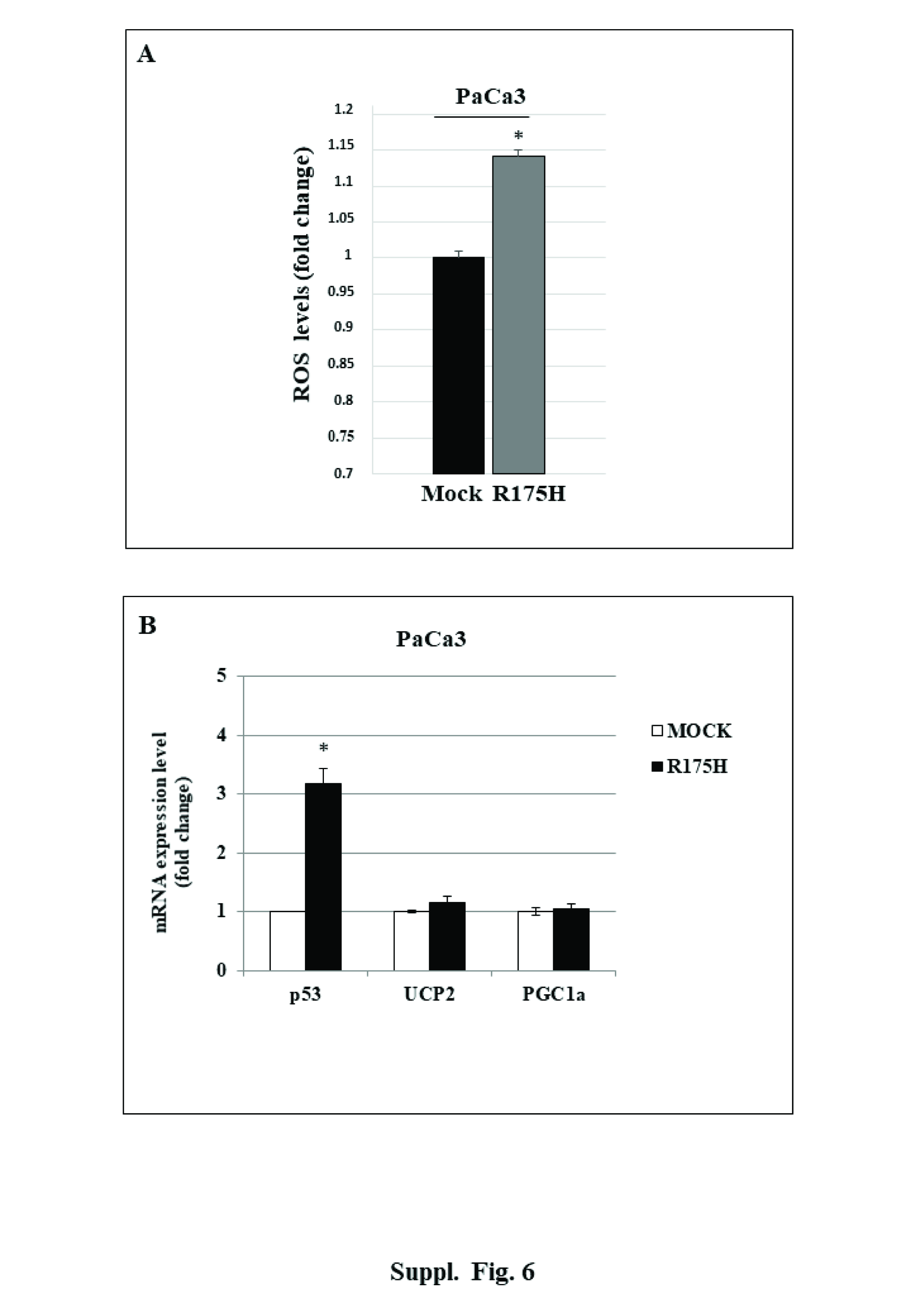

Supplement: Supplementary file 6 — Supplementary Figure 6 [file 41416_2018_288_MOESM6_ESM.tif]
