# Supplementary material for: Mutant p53 blocks SESN1/AMPK/PGC-1α/UCP2 axis increasing mitochondrial O2ˉ· production in cancer cells
Source: Br J Cancer. 2018 Oct 15;119(8):994–1008. doi: 10.1038/s41416-018-0288-2 (PMC6203762; doi:10.1038/s41416-018-0288-2)
Supplement: Supplementary file 7 — Legends to Supplementary Figures [file 41416_2018_288_MOESM7_ESM.docx]

**Legends to Supplementary Figures**

**Supplementary Figure 1 - Mutant p53 cells show higher ROS as compared to WT-p53 cells.** The endogenous basal level of ROS in cancer cells carrying WT or mutant p53 was analyzed using the incorporation of the DCF probe. Cells were seeded in 96-well plates, incubated overnight, and stained with DCF as described in Material and Methods. * p<0.05 mutant p53 cells vs WT-p53 cells.

**Supplementary Figure 2 - Kaplan-Meier curves of TTFT for CLL patient subgroups defined by different mutational status of the *TP53* gene.** The *TP53* gene mutational status of CLL patients analyzed is reported in Supplementary Table 2. Statistical analysis was performed using the Student’s test. Data are expressed as mean ± SEM. *: P<0.05.

**Supplementary Figure 3 - Mutant p53 inhibits expression of SESN1 and SESN2 mRNAs.** Panc1 mutR273H p53 and AsPC1-p53 null cells were transfected with pRSuperp53 (or its negative control) and with the vector for mutant p53 ectopic expression (or its mock vector), respectively. Gene expression analysis of SESN1 and SESN2 was performed by RT-qPCR and normalized to GAPDH mRNA. * p<0.05.

**Supplementary Figure 4 - AICA-R induces P-(Thr172)AMPK.** Panc1 mutR273H p53 cells were treated with 1 mM AICA-R for 72 h. Western blotting was performed using 50 μg of whole cell extracts, probed with the indicated antibodies and quantified with ImageJ software. * p<0.05.

**Supplementary Figure 5 - Control of UCP2 or p53 gene modulation.** Panc1 mutR273H p53 and AsPC1-p53 null cells were transfected with pRSuperp53, siUCP2 and vector for mutant p53 ectopic expression respectively and their controls. Gene expression analysis of UCP2 and p53 was performed by RT-qPCR and was normalized to GAPDH mRNA. * p<0.05.

**Supplementary Figure 6 - PGC-1α/UCP2 axis is not regulated by endogenous wild-type p53.** (A) PaCa3 cells were seeded in 96-well plates, incubated overnight, and transfected with the pRSuper-p53 vector for expression of R175H mutant p53 or its negative mock control. DCF fluorescence intensity was analyzed by a multimode plate reader. * p<0.05. (B) PaCa3 cells were transfected for 48 h with the pRSuper-p53 vector for expression of R175H p53 mutant or its relative negative control. Gene expression analysis of p53, UCP2 and PGC-1α was performed by RT-qPCR and normalized to GAPDH mRNA. * p<0.05.
