# Supplementary material for: Mutant p53 blocks SESN1/AMPK/PGC-1α/UCP2 axis increasing mitochondrial O2ˉ· production in cancer cells
Source: Br J Cancer. 2018 Oct 15;119(8):994–1008. doi: 10.1038/s41416-018-0288-2 (PMC6203762; doi:10.1038/s41416-018-0288-2)
Supplement: Supplementary file 8 — Supplementary Table 1 [file 41416_2018_288_MOESM8_ESM.docx]

**Supplementary Table 1.** Sequences of primers used for qPCR analysis

| **Genes** | **Primer sequences** |
| --- | --- |
| ***PGC1α*** | for: 5' tgactggcgtcattcaggag 3’ |
|  | rev: 5' ccagagcagcacactcgat 3’ |
|  |  |
| ***UCP2*** | for: 5' ctcctgaaagccaacctcat 3’ |
|  | rev: 5' cccaaaggcagaagtgaagt 3’ |
|  |  |
| ***TP53*** | for: 5' ggcccacttcaccgtactaa 3’ |
|  | rev: 5' gtggtttcaaggccagatgt 3’ |
|  |  |
| ***SESN1*** | for: 5' ggacgaggaacttggcatta 3’ |
|  | rev: 5' atgcatctgtgcgtcttcac 3’ |
|  |  |
| ***SESN2*** | for: 5' gcctgctacccagagaagac 3’ |
|  | rev: 5' cctccaggagcagcaagtt 3’ |
|  |  |
| ***GAPDH*** | for: 5’ atcagcaatgcctcctgcac 3’ |
|  | rev: 5’ tggtcatgagtccttccacg 3’ |
|  |  |
| **mtDNA short fragment** | for: 5’ cagcacaaagtcctgtggaa 3’ |
|  | rev: 5’ cgaagaggtaagatcatctggt 3’ |
|  |  |
| **mtDNA long fragment** | for: 5’ cctgaaacttcaatgccaaa 3’ |
|  | rev: 5’ cgaagaggtaagatcatctggt 3’ |
