# Supplementary material for: Mutant p53 blocks SESN1/AMPK/PGC-1α/UCP2 axis increasing mitochondrial O2ˉ· production in cancer cells
Source: Br J Cancer. 2018 Oct 15;119(8):994–1008. doi: 10.1038/s41416-018-0288-2 (PMC6203762; doi:10.1038/s41416-018-0288-2)
Supplement: Supplementary file 9 — Supplementary Table 2 [file 41416_2018_288_MOESM9_ESM.docx]

**Supplementary Table 2.** *TP53* gene mutations in CLL patients

| Patient | Exon 4 | Exons 5-6 | Exon 7 | Exons 8-9 | Mutation type |
| --- | --- | --- | --- | --- | --- |
| 0134 | WT | p.L201fs*15 c.602delT | WT | WT | Frameshift |
| 0144 | WT | p.T211fs*5 c.632_633insT | WT | WT | Frameshift |
| 0040 | WT | p.E180D c.540G>T +  p.R209fs*6 c.626_627delGA | WT | WT | Missense +  Frameshift |
| 0035 | WT | p.H179Q c.536A>G | WT | WT | Missense |
| 0059 | WT | p.H179R c.536A>G | WT | WT | Missense |
| 0181 | WT | p.Y220C c.659A>G | WT | WT | Missense |
| 0012 | WT | WT | p.R248Q c.743G>A | WT | Missense |
| 0037 | WT | WT | p.G245D c.734G>A | WT | Missense |
| 0084 | WT | WT | p.C242S c.725G>C | WT | Missense |
| 0161 | WT | WT | p.G244C c.730G>T | WT | Missense |
